# 3D Bioprinted Breast Cancer‐Stroma Model with Tailored Migration‐Permissive Bioink Reveals Impact of Adipose‐Derived Stromal Cells on Cancer Cell Migration and Invasion Dynamics

**DOI:** 10.1002/adhm.71353

**Published:** 2026-07-26

**Authors:** Sabrina Stecher, Joachim Schenk, Alessandro Cianciosi, Katherina Hemmen, David Böhringer, Franziska Dusi, Jessica Faber, Philipp Stahlhut, Benedikt Gantert, Emilie Rouenhoff, Annika Gmöhling, Tessa Lühmann, Jörg Teßmar, Jürgen Groll, Silvia Budday, Tomasz Jüngst, Ben Fabry, Katrin G. Heinze, Petra Bauer‐Kreisel, Torsten Blunk

**Affiliations:** ^1^ Department of Trauma Hand, Plastic and Reconstructive Surgery University Hospital Würzburg Würzburg Germany; ^2^ Rudolf Virchow Center Center for Integrative and Translational Bioimaging University of Würzburg Würzburg Germany; ^3^ Chair for Functional Materials in Medicine and Dentistry at the Institute of Functional Materials and Biofabrication University of Würzburg and Bavarian Polymer Institute Würzburg Germany; ^4^ Department of Physics University of Erlangen‐Nürnberg Erlangen Germany; ^5^ Institute of Continuum Mechanics and Biomechanics University of Erlangen‐Nürnberg Fürth Germany; ^6^ Institute of Pharmacy and Food Chemistry University of Würzburg Würzburg Germany

**Keywords:** bioprinting, breast cancer, cancer, invasion, live‐cell imaging, migration

## Abstract

In breast cancer, local invasion of cancer cells into surrounding tissue marks the first step of metastasis. However, to elucidate the impact of cells from the tumor microenvironment on this process, advanced 3D migration models are still urgently needed. To enable migration and invasion studies in a fully 3D bioprinted tumor‐stroma model, a migration‐permissive bioink composed of methacrylated collagen type I and thiolated hyaluronic acid with low polymer content is developed. In a printed co‐culture model comprising metastatic breast cancer cells (MDA‐MB‐231) and adipose‐derived stromal cells (ASCs), real‐time single‐cell tracking reveals that ASCs in the stromal compartment profoundly promote migration and invasion dynamics of individual tumor cells. This is reflected by increased speed, migration distance, and invasion into the stroma, and is accompanied by collagen remodeling and a shift in tumor cell morphology. A correlation between tumor cell morphology and migration speed is evident, which is modulated by ASCs. A highly motile and invasive subset of tumor cells is significantly enhanced in the presence of ASCs. These insights into the influence of ASCs on the heterogeneity of breast cancer cells in terms of their migratory behavior may inform the development of more specific and effective treatment options for metastatic breast cancer.

## Graphical abstract

A migration‐permissive bioink composed of methacrylated collagen type I and thiolated hyaluronic acid enables 3D bioprinting of breast cancer‐stroma models. Single‐cell tracking reveals that adipose stromal cells enhance tumor cell motility and stromal invasion, accompanied by collagen remodeling, and a shift in tumor cell morphology. This bioprinted platform offers a physiologically relevant, adaptable framework to dissect stroma‐driven tumor cell invasion.

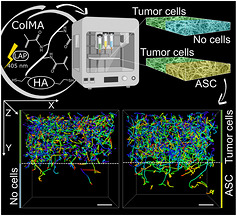

## 1 Introduction

Local invasion of cancer cells into the surrounding tissue represents the first critical step in the metastatic cascade and the basis for subsequent dissemination of cancer cells to distant sites [1, 2]. During invasion, tumor cells display remarkable phenotypic plasticity, adapting their shape and mode of motility in response to physical constraints and biochemical cues from the tumor microenvironment (TME) [3, 4, 5].

In breast cancer, the abundant adipose tissue within the TME contributes to the invasive process as adipose‐derived stromal cells (ASCs) and adipocytes have been shown to enhance the migratory potential of malignant cells [6]. However, these findings are mostly derived from classical 2D co‐culture wound healing and transwell migration assays. While these approaches can provide some valuable insights into the influence of paracrine signaling on cancer cell migration, they do not adequately capture the multifaceted interaction between cancer cells and the TME in a 3D context [7, 8, 9, 10]. Consequently, advanced 3D models that closely mimic the TME are imperative for understanding to what extent stromal cells modulate breast cancer cell migratory behavior and dynamics, particularly migration speed and directionality, which are essential for effective stromal invasion.

3D extrusion bioprinting has emerged as a promising approach in tissue engineering for creating complex, cell‐laden 3D in‐vitro models. By allowing the precise and reproducible deposition of multiple cell types and matrix materials in specific patterns, this technique is particularly well‐suited for mimicking the architectural complexity of native tissue and cancer heterogeneity [11]. However, a major challenge of the field remains the development of extrusion‐printable bioinks that are suitable for cancer cell migration studies. Collagen type I represents a natural ECM component in the TME that has been used as a standard material in migration studies in non‐bioprinted setups, as it provides a number of required properties, including sufficient cell adhesion sites (e.g., integrin binding sites), and enzymatic degradability (allowing cells to remodel their environment). Furthermore, for non‐printed hydrogels, the concentration can be adjusted to yield a moderate stiffness and viscoelasticity (giving cells sufficient anchorage but also allowing them to deform the matrix during migration), as well as a medium pore size (as both excessively small and large pores hinder cell migration) [12, 13, 14, 15, 16, 17]. For the use as a bioink in extrusion bioprinting, collagen type I has been chemically modified, e.g., in a methacrylated form (ColMA), and utilized in various tissue engineering applications. However, printability and shape fidelity after printing were ensured by a rather high polymer content [18, 19, 20, 21, 22, 23], which would substantially compromise cell migration and thus considerably limit the applicability for dynamic migration analysis [24, 25]. Consequently, ColMA‐based bioinks for extrusion‐printing that readily allow tumor cell migration and are suitable for such cell tracking analysis still need to be demonstrated.

Overall, to date, fully 3D extrusion‐bioprinted tumor‐stroma models investigating cancer cell migration and invasion remain limited [23, 26, 27]. The studies mainly focused on drug efficacy, and although cancer cell invasion was reported, neither study incorporated cell tracking techniques for detailed analysis of cell migration [23, 26, 27]. Other models employed a modular fabrication approach, where cancer cell migration was confined to the pre‐defined cast compartments, a limitation that distinctly restricts geometric complexity [28, 29, 30]. The modular models established by Meng et al. and DeLorenzi et al. used bioreactor systems to examine the effects of vasculature and growth factors on lung and skin cancer cell migration, respectively, but again did not quantify dynamic migration metrics [28, 29]. Our laboratory previously developed modular models for precise monitoring of cancer cell migration influenced by adjacent adipose microtissues. However, collagen I was only used in a non‐bioprinted form, and the hyaluronic acid‐based bioink employed for the stromal compartment failed to support cell migration, thereby limiting the analysis to paracrine interactions and preventing the assessment of invasive behavior [30].

In this study, we developed a bioink based on low‐concentration collagen type I and hyaluronic acid that is extrusion‐printable and, at the same time, readily allows tumor cell migration. Exploiting this bioink, we engineered a new fully 3D bioprinted compartmentalized breast cancer‐stroma model. Using high‐resolution live‐cell imaging and single‐cell tracking, we gained novel insights into the impact of ASCs on the dynamics and heterogeneity of breast cancer cell migration and invasion.

## 2 Results and Discussion

### 2.1 Development of ColMA‐Based Bioinks

Extrusion‐based bioprinting is a powerful technique with great potential for the development of disease models [11, 31]. However, printability itself often requires bioinks with high polymer concentrations and high viscosity, while for adequate cellular behavior, in general, low biomaterial content and high porosity are considered favorable [24, 32]. This applies especially to cell migration and invasion processes, which is why printable materials for migration studies are largely lacking. Therefore, we established a new 3D printable bioink that is well applicable to investigate cell migration and invasion.

As illustrated in Figure 1A, the developed bioink consists of biopolymers that account for a significant proportion of cancerous ECM [33, 34] and are used here as functionalized derivatives, namely methacrylated collagen type I (ColMA) and thiolated hyaluronic acid (HA‐SH). Exposure to UV light initiates a radical‐mediated thiol‐ene reaction in the presence of the photoinitiator lithium phenyl‐2,4,6‐trimethylbenzoylphosphinate (LAP). Thereby, the methacrylate groups of ColMA, as the main component, react with the thiol groups of the crosslinker HA‐SH, forming covalent thioether bonds. Simultaneously, methacrylate groups within ColMA undergo additional radical polymerization, leading to stable and easy‐to‐handle constructs. To prevent premature thermo‐induced collagen polymerization and ensure controlled collagen fiber formation, which is crucial for maintaining the structural integrity necessary for cell adhesion [35], bioink preparation and printing are carried out under pH and temperature control. Different formulations of the ColMA/HA‐SH bioink with overall low polymer content were systematically tested to optimize printability by varying the ColMA concentration (ranging from 1 to 5.5 mg mL^−1^) while keeping the HA‐SH and LAP concentrations constant at 1 and 0.8 mg mL^−1^, respectively. Three complementary printing tests, the grid structure, filament fusion, and 4‐angled pattern test, showed that increasing the ColMA concentration improved the bioink's printing resolution (Figure 1B and Figure S1). This was confirmed by evaluating the strand intersection diagonal of printed grids and the filament width (Figure 1C,D and Figure S1B). Although the bioink was printable over a large range of ColMA concentrations, only bioink formulations with a ColMA concentration of ≥ 2.5 mg mL^−1^ were mechanically stable after photocrosslinking. It has to be emphasized that printability at such low ColMA concentrations as used here was only achievable due to the presence of HA‐SH as crosslinker; when HA‐SH was omitted from the bioink, the resulting grid structure was distinctly compromised (Figure S2). A low polymer content is particularly favorable for cellular movement, as it reduces mechanical constraints and enhances the ability of cells to migrate. At the same time, using ECM‐derived polymers as the primary bioink components provides a biologically relevant microenvironment rich in biochemical cues that favor cell adhesion and migration [24]. For that reason, ColMA‐based bioinks recently gained considerable attention. Several studies have sought to optimize the printability of ColMA‐based bioinks by incorporating additives such as GelMA [19], heprasil [20, 21], gelatin [18], or HA‐SH [22]. However, the formulations designed for extrusion‐based bioprinting already employed a high ColMA content, and the integration of additives has further elevated the total polymer concentration [18, 19, 20, 21, 22]. By contrast, formulations with lower polymer concentrations (in the range of 3–6 mg mL^−1^) have only been successfully processed using FRESH (Freeform Reversible Embedding of Suspended Hydrogels) printing, which utilizes a support bath to maintain structural fidelity during bioprinting [36, 37, 38, 39]. These ColMA bioinks have been used for a variety of different tissue constructs; however, to date, such inks have not been used in live‐cell migration studies. With our new low‐concentration bioink formulations, we enable the use of ColMA in bioprinted constructs that are well‐suited for detailed investigation of cancer cell migration.

**FIGURE 1:**
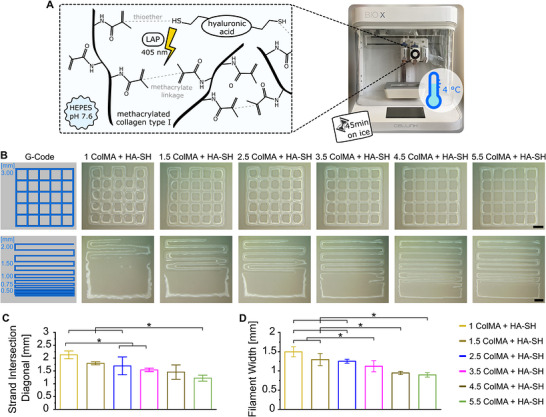
Crosslinking mechanism and evaluation of printability of collagen type I‐based bioinks. (A) Neutralized methacrylated collagen type I (ColMA) is mixed with thiolated hyaluronic acid (HA‐SH) and the photoinitiator LAP, which are both dissolved in HEPES (154 mM, pH 7.6). The precursor solution is incubated for 45 min on ice prior to printing. Subsequent crosslinking at 405 nm leads to stable constructs. (B) Grid structure and filament fusion test of different bioink formulations (from left to right: 1/1.5/2.5/3.5/4.5/5.5 mg mL^−1^ ColMA + 1 mg mL^−1^ HA‐SH + 0.8 mg mL^−1^ LAP). Bioinks were printable over a wide range of ColMA concentrations, whereby printing resolution increased with increasing ColMA concentration and only printed constructs ≥ 2.5 mg mL^−1^ were mechanically stable after photocrosslinking. All printing tests were performed using a 330 µm blunt steel nozzle. Scale bar: 3 mm. (C) Quantification of the strand intersection diagonal of printed grids and (D) of the filament width in printed filament fusion tests. Data are presented as mean ± SD (n = 3–10). Significances were calculated using one‐way ANOVA followed by Tukey–Kramer test. Statistically significant differences are indicated by ^*^ (*p* < 0.05).

### 2.2 Rheological and Mechanical Characterization of ColMA/HA‐SH Bioinks

To further evaluate the suitability of the different bioink formulations for extrusion‐based bioprinting, we investigated the rheological properties of the bioink precursor solutions. In shear rate sweep tests, the viscosity profiles of selected formulations (2.5, 3.5, 5.5 mg mL^−1^ ColMA with 1 mg mL^−1^ HA‐SH, and 2.5 mg mL^−1^ ColMA alone) were determined (Figure 2A). They all exhibited decreasing viscosity with increasing shear rate, demonstrating shear thinning behavior, which is favorable for extrusion‐based 3D printing to ensure smooth material deposition while preserving shape fidelity post‐printing [40]. Furthermore, a photorheological characterization was performed to simulate the light‐induced crosslinking step (Figure 2B). Time sweeps of the investigated bioink formulations showed a distinct increase in the storage modulus (G′) after light exposure, with a direct correlation between ColMA concentration and G′. As expected, only the formulations containing HA‐SH as crosslinker showed a sol‐to‐gel transition (G′ > G″ after light exposure) (Figure 2B). Frequency sweep measurements showed that for the ColMA/HA‐SH inks, G′ remained higher than G″ across the entire frequency interval, which is indicative of a solid‐like network (Figure 2C). In contrast, the HA‐SH‐free formulation exhibited an inverted trend (G′ < G″) over the same range, consistent with liquid‐like behavior (Figure S3). The soft nature of these bioinks was also demonstrated through uniaxial compression tests. The stress‐stretch curves showed the typical nonlinear viscoelastic behavior of collagen (Figure 2D), which has been found to be critical for cell migration [41, 42, 43]. Expectedly, the maximal nominal stress increased with increasing ColMA concentration (Figure 2E). To determine the corresponding apparent Young's moduli of the unconditioned mechanical response, hyperelastic material modeling of cyclic compression tests was conducted (Figure 2F). This yielded Young's moduli of 153, 257, and 422 Pa for formulations with 2.5, 3.5, and 5.5 mg mL^−1^ ColMA, respectively (Figure 2G,H). The low polymer concentrations and crosslinking conditions used in this study resulted in soft bioinks with elastic moduli matching those reported in cell migration studies for non‐printable collagen I hydrogels [14, 43, 44]. Thus, this mechanical profile renders the developed bioink formulations particularly well‐suited for investigating invasive cell behavior, now enabled for 3D bioprinted models.

**FIGURE 2:**
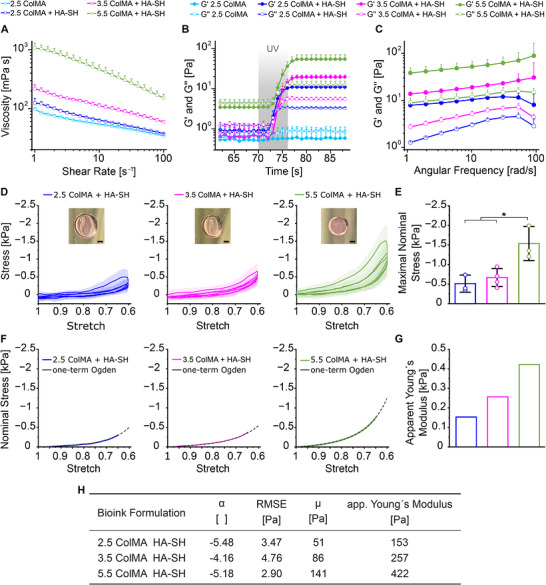
Rheological, photorheological, and mechanical characterization of ColMA‐based bioinks. (A) Shear rate sweep test before photocrosslinking showing the viscosity profile of 2.5, 3.5, and 5.5 mg mL^−1^ ColMA + 1 mg mL^−1^ HA‐SH, and 2.5 mg mL^−1^ ColMA only bioinks. (B) Time sweep measurements before, during, and after photocrosslinking to study the sol‐to‐gel transition of the bioinks. (C) Frequency sweeps of the different ink formulations after photocrosslinking. All rheological data are presented as mean ± SD (n = 3). (D) Cyclic compression behavior of 2.5, 3.5, and 5.5 mg mL^−1^ ColMA/HA‐SH hydrogels up to a maximum strain of 40 %. Scale bar: 1 mm. (E) Corresponding average maximum nominal stresses. Data are presented as mean ± SD (n = 3–4). Significances were calculated using one‐way ANOVA followed by Tukey–Kramer tests for multiple comparisons (^*^ *p* < 0.05). (F) Modified one‐term Ogden model calibrated with the average unconditioned mechanical response of ColMA/HA‐SH hydrogels during cyclic compression up to a strain of 35 %. (G) Corresponding apparent Young's moduli of the unconditioned mechanical response obtained from fitting the modified one‐term Ogden model to the first cycle of cyclic compression data up to a strain of 35 %. (H) Table of the nonlinearity parameter α, classical shear modulus µ, corresponding root mean square error RMSE, and calculated apparent Young's modulus of the unconditioned mechanical response of ColMA/HA‐SH hydrogels obtained from fitting the modified one‐term Ogden model to the first cycle of cyclic compression data up to a maximum strain of 35 %.

### 2.3 Ultrastructure of ColMA/HA‐SH Bioinks

For comprehensive characterization, the ultrastructural properties of the ColMA/HA‐SH bioinks were analyzed. Scanning electron microscopy (SEM) images showed a comparable nanoscale ultrastructure in the polymer networks of printed and cast ColMA/HA‐SH constructs, indicating that the printing process preserved collagen fibrillation (Figure 3A). Quantitative analysis confirmed the consistency in fibril morphology, with mean fibril diameters of 42.42 ± 2.41 nm and 41.30 ± 1.11 nm for printed and cast constructs, respectively (Figure 3B). These findings are consistent with prior observations that, despite methacrylation, ColMA predominantly maintains its native quaternary structure upon polymerization, and that photocrosslinking does not alter its fibrillar morphology [37, 45]. The presence of typical collagen fibers, which provide integrin‐binding sites essential for regulating cell behavior, including cell migration [46, 47], represents a key advantage of the ColMA/HA‐SH bioink over alternatives such as alginate [48] or hyaluronic acid‐based bioinks [30, 49]. To visualize the collagen fiber network of different bioink formulations without dehydrating the hydrogels, we used confocal reflection microscopy. Expectedly, an increase in network density with increasing ColMA concentration was observed (Figure 3C). The distribution of the collagen network was also evident in the 3D reconstructions presented in the Supplement Video S1. With an average pore diameter of ∼10 µm, which is in the range of the cell diameter [50], the ColMA/HA‐SH hydrogels fulfill yet another prerequisite for facilitating cell migration.

**FIGURE 3:**
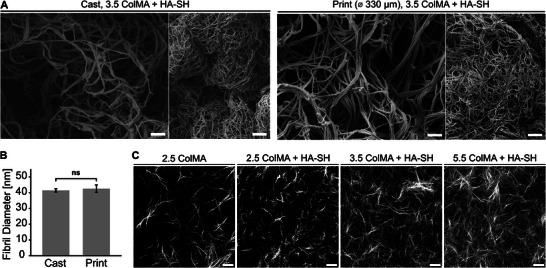
Analysis of the ultrastructure of ColMA/HA‐SH hydrogels. (A) Scanning electron microscopy images of cast and printed ColMA/HA‐SH hydrogels. Scale bar: 400 nm and 2 µm. (B) Corresponding quantification of fibril diameter (n = 3, 50 fibers per n, mean ± SD, significances were calculated using an unpaired Student´s *t*‐test). (C) Confocal reflection microscopy images depicting the native collagen fiber network of different bioink formulations. For visualization, images were contrast‐enhanced and gamma‐corrected. Scale bar: 20 µm.

### 2.4 Cell Viability in ColMA/HA‐SH Bioink Post‐Printing

Live/dead staining of MDA‐MB‐231 breast cancer cells embedded in ColMA/HA‐SH ink revealed high cell viability post‐printing with an overall cell survival rate of around 90 %, with no significant differences between different nozzle sizes (Figure 4A,B). Even though the pneumatic extrusion of the cell‐laden ink during printing is known to cause shear stress and thus possible cell damage [51], the results confirmed that the printing conditions were suitable for maintaining cell viability, further validating the bioink as an effective cell carrier for bioprinting applications.

**FIGURE 4:**
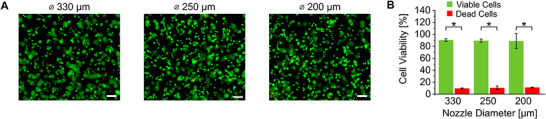
Analysis of cell viability post printing (3.5 mg mL^−1^ ColMA/HA‐SH). (A) Live/dead staining of MDA‐MB‐231 cells after printing using a 330, 250, or 200 µm blunt steel nozzle. Living cells were stained with calcein‐AM (green) and dead cells with EthD‐III (red). Scale bar: 200 µm. (B) Quantification of respective MDA‐MB‐231 cell viability (n = 3, mean ± SD, significances were calculated using a two‐way RM ANOVA followed by Tukey‐Kramer post hoc test (^*^ *p* < 0.05)).

### 2.5 Cancer Cell Migration in ColMA/HA‐SH Bioink in Spheroid Invasion Assays

The general suitability of our ColMA/HA‐SH bioinks for cell migration studies was evaluated in spheroid invasion assays. Two distinct ColMA concentrations were used, 2.5 or 3.5 mg mL^−1^ ColMA (Figure 5A). In both bioink formulations, tumor cells were able to invade the surrounding hydrogel, with a higher invasion capacity in 2.5 mg mL^−1^ ColMA (Figure 5B,C and Figure S4).

**FIGURE 5:**
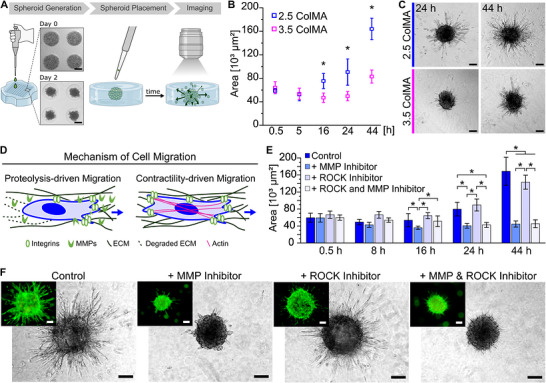
Analysis of cell migration from MDA‐MB‐231 spheroids in ColMA/HA‐SH bioink. (A) Scheme of the MDA‐MB‐231 spheroid invasion assay. Cancer cell spheroids were generated using agarose micro‐molds and a two‐step centrifugation protocol. Subsequently, spheroids were embedded in ColMA/HA‐SH hydrogels, and their migration into the surrounding matrix was observed over time. (B) Quantification of MDA‐MB‐231 spheroid area embedded in 2.5 or 3.5 mg mL^−1^ ColMA/HA‐SH (n = 6–10, significances were calculated using unpaired two‐sample Welch´s *t*‐test) and (C) representative phase contrast images. Scale bar: 100 µm. (D) Scheme of the two investigated mechanisms of cell migration: proteolysis and contractility‐driven migration. (E) Quantification of cell‐covered areas over time with MMP inhibitor, ROCK inhibitor, both treatments, or no treatment (control) (n = 8–12, significances were calculated using one‐way ANOVA followed by Tukey‐Kramer post hoc test) and (F) respective phase contrast images at *t* = 44 h. Inserts: Live/dead staining. Living cells were labeled with calcein‐AM (green) and dead cells with EthD‐III (red). Scale bar: 100 µm. All data are presented as mean ± SD (*p* < 0.05 is indicated by ^*^).

To further investigate the mechanism of cell migration within the 2.5 mg ml^−1^ ColMA bioink, we added a broad‐spectrum MMP inhibitor (Batimastat) to suppress protease‐driven migration and/or a ROCK inhibitor (Y‐27632) to hinder contractility‐driven cell migration (Figure 5D). As shown in Figure 5E,F, MMP inhibition drastically hindered cell migration (no increase of spheroid area), an effect also seen when both the ROCK and MMP inhibitors were applied together. In contrast, ROCK inhibition alone exhibited a significantly less pronounced effect on migration ability. Live/dead staining confirmed cell viability across all four conditions (Figure 5F). These observations were recapitulated in 3.5 mg mL^−1^ ColMA/HA‐SH hydrogels with a slightly time‐delayed response (Figure S5), indicating the pivotal role of proteolytic processes for MDA‐MB‐231 cell migration in ColMA/HA‐SH constructs. Further, the results align with previous observations that the invasive capacity of breast cancer cells in collagen gels depends highly on MMP‐dependent ECM remodeling [7, 52]. Overall, these findings underscore the suitability of our newly established bioink as a functional collagen analog for cell migration studies. Complementary gene expression analysis (Figure S6) showed that embedded cells expressed a variety of MMPs, including key cancer‐associated MMPs such as MMP‐1, MMP‐7, MMP‐9, MMP‐11, and MMP‐14 [53]. Notably, MMP‐1, which is important for collagen type I degradation and has been shown to be linked to tumor invasiveness [54], was significantly up‐regulated at higher ColMA concentrations, with expression increasing over time. Similarly, MMP‐1 protein levels also increased over time, though higher ColMA concentrations led to reduced protein secretion (Figure S6B). This trend was further confirmed by measuring the general protease activity of the supernatant (Figure S6C) and was in line with previous findings [25]. The observed difference in MMP‐1 mRNA and protein expression could be due to post‐transcriptional mechanisms, such as mRNA stability and translation regulation, as previously shown [55, 56].

### 2.6 Cancer Cell Migration as a Function of Stromal Cells in Spheroid Invasion Assays

To investigate whether and to what extent cancer cell migration can be modulated by stromal cells within the ColMA/HA‐SH bioink, MDA‐MB‐231 spheroids were embedded in constructs containing either ASCs, adipocytes, or no cells as a control (Figure 6A). Selective quantification of cancer cell migration was achieved using MDA‐MB‐231 cells that express cytosolic GFP. The migratory behavior of these cells was verified to be unaffected by GFP expression (Figure S7). Both stromal cell types markedly increased cancer cell migration after 44 h, compared to the cell‐free control (Figure 6B). Even under MMP (Batimastat) or ROCK inhibition (Y‐27632), the invasion of MDA‐MB‐231 cells was significantly increased by both stromal cell types (Figure 6B and Figure S8).

**FIGURE 6:**
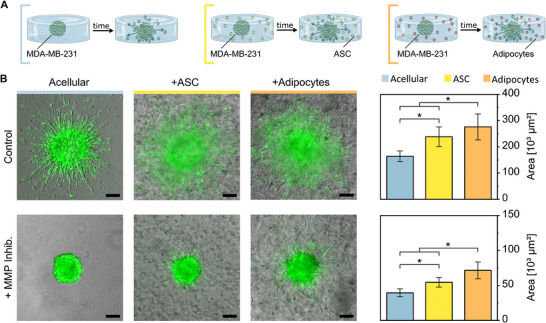
Analysis of MDA‐MB‐231 cell migration in spheroid invasion assays in dependence on ASCs or adipocytes. (A) Scheme of experimental setup. ASCs, adipocytes, or no cells were embedded in 2.5 mg mL^−1^ ColMA/HA‐SH hydrogel. Prior to final crosslinking of the gel, GFP‐expressing MDA‐MB‐231 spheroids were placed into the center of the hydrogel. Constructs were either cultured in the presence of the MMP inhibitor Batimastat or in cell culture media only. (B) Phase contrast/fluorescence merge images of MDA‐MB‐231 spheroids and ASCs, adipocytes, or no surrounding cells embedded in ColMA/HA‐SH hydrogel and respective quantification of the occupied area after 44 h in culture. Data are presented as mean ± SD (n = 10–15). Significances were calculated using one‐way ANOVA followed by Tukey‐Kramer post hoc test (^*^ *p* < 0.05). Scale bar: 100 µm.

These results demonstrate that the migration of MDA‐MB‐231 cells in the ColMA/HA‐SH bioink can be modulated by adjacent stromal cells, highlighting the pro‐migratory roles of ASCs and adipocytes [7, 30] and suggesting that stromal cells promote cancer cell migration through mechanisms that are not solely dependent on either MMP or ROCK activity.

### 2.7 3D Bioprinted Tumor‐Stroma Model for Analyzing Migration and Invasion in Response to ASCs

#### 2.7.1 Development of 3D Bioprinted Tumor‐Stroma Model

We demonstrated that the newly developed bioink permits extrusion‐based bioprinting at low polymer concentrations, promotes cell migration, and facilitates the investigation of stromal influences on cancer cell migration. In addition, the ColMA/HA‐SH bioink provides favorable optical properties, including low light scattering and minimal autofluorescence that support high‐resolution live‐cell imaging. These features enabled the engineering of a compartmentalized, fully 3D bioprinted tumor‐stroma model to systematically investigate the impact of ASCs on cancer cell migration and invasion dynamics on a single‐cell level. The co‐culture model was completely printed with ColMA/HA‐SH bioink and comprised two spatially distinct compartments: a tumor compartment containing MDA‐MB‐231 cells, which stably expressed cytosolic GFP, and a stromal compartment containing either ASCs (ASC compartment) or no cells (acellular compartment). The two stromal conditions differed solely regarding the presence of ASCs, with comparable collagen fiber architecture and Young's modulus (Figure S9). Thus, this defined spatial architecture allowed us to examine cancer cell migration in response to ASCs, and, to the best of our knowledge, enabled the first detailed investigation of cancer cell invasion into the stromal compartment within 3D bioprinted models using quantitative migration tracking. Figure 7A provides an overview of the experimental workflow. GFP‐expressing MDA‐MB‐231 cells, ASCs, or no cells were resuspended in bioinks with 3.5 mg mL^−1^ ColMA, transferred into cartridges, and kept on ice. Using three printheads, we generated both types of tumor‐stroma models within the same setup (i.e., stromal compartment either with ASCs or acellular). Models were printed directly into silicon chambers that were mounted on coverslips to ensure optimal imaging. Printed constructs were imaged over 95 h, capturing brightfield and fluorescence z‐stacks. After image deconvolution and segmentation, we tracked cell migration in x/y/z‐direction using the autoregressive motion model [57]. Figure 7B presents exemplary segmentation masks of the tumor‐stroma models over time that were used for quantitative tracking of cancer cells. Of the resulting tracks, we only used those with track lengths exceeding 20 µm and with a continuous track duration of more than 24 h for computing key migration parameters: track length, migration speed, cell sphericity, and displacement toward the stromal compartment. For illustration, tracks fulfilling the aforementioned requirements are shown in Figure 7C.

**FIGURE 7:**
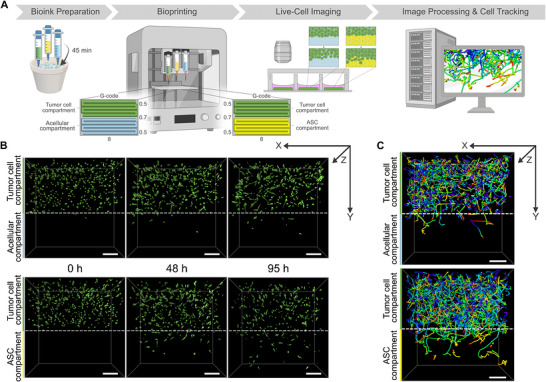
Multi‐dimensional (x, y, z, time) imaging of MDA‐MB‐231 cancer cell migration in 3D bioprinted tumor‐stroma models. (A) Graphical overview of the workflow generating compartmentalized tumor‐stroma models. The tumor compartment contained GFP‐expressing MDA‐MB‐231 cells, while the stromal compartment contained either ASCs (ASC compartment) or remained cell‐free (acellular compartment). Z‐stacks of printed models were captured at 1 h intervals over a period of 95 h. Following deconvolution and segmentation, cancer cells were tracked in three dimensions (x/y/z). (B) Exemplary segmentation masks of migrating MDA‐MB‐231 cells in 3D printed tumor‐stroma models of both groups (ASC and acellular) over time, and (C) respective continuous trajectories longer than 24 h of both groups used for subsequent analysis of track length, speed, sphericity, and displacement toward stroma. The dashed line indicates the interface between the two compartments. Scale bar: 200 µm.

#### 2.7.2 ASC‐Dependent Increase of Breast Cancer Cell Migration Speed and Distance

Quantification of track lengths revealed that cancer cells migrated distinctly farther in the presence of ASCs. More specifically, cancer cells in constructs with the ASC compartment migrated an average distance of 475 ± 13 µm, while cancer cells in constructs with an acellular stromal compartment migrated an average distance of 411 ± 18 µm (Figure 8A). Interestingly, in both conditions, most track lengths clustered around the modes, and a small fraction of cells migrated over larger distances (Figure 8A). Analysis of the track length of the longest 10 % further confirmed the tendency of cancer cells to migrate farther in the presence of ASCs than in the absence of stromal cells (Figure 8B). Our observations are consistent with reports from 3D microfluidic co‐culture systems, where cancer‐associated fibroblasts similarly increased the migration distance of triple‐negative SUM‐159 breast cancer cells [58, 59].

**FIGURE 8:**
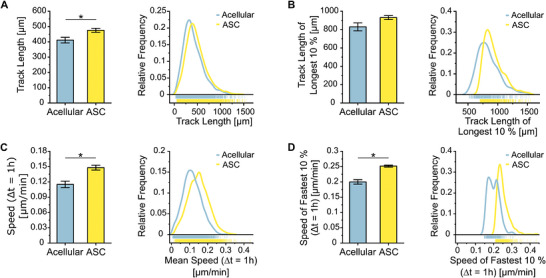
Migration parameters of MDA‐MB‐231 cells in response to ASCs in the 3D bioprinted tumor‐stroma model. (A) Total track length. Left: average track length. Right: track length distributions as density curves with a rug plot indicating individual data points. (B) Track length of longest 10 %. (C) Average speed (Euclidean distance between all x/y/z‐positions of the same cell of consecutive frames, divided by the time interval ∆t = 1 h averaged over the observation period for each cell trajectory) and corresponding distributions as density curves with rug plots indicating individual data points. (D) Speed of the fastest 10 %. (A–D) Data are presented as mean of mean ± SE (n = 9, whereby each n comprised ∼450 tracks). Significances were calculated using an unpaired Student´s *t*‐test. *p* < 0.05 is indicated by ^*^.

To further assess migratory behavior, we quantified the average speed of the cancer cells. As shown in Figure 8C, the presence of ASCs led to a significant increase in average speed, from 0.115 ± 0.006 µm min^−1^ to 0.148 ± 0.005 µm min^−1^. This trend persisted when comparing the fastest 10 % tracks: cancer cells in the presence of ASCs had an average speed of 0.252 ± 0.003 µm min^−1^, while in the absence of stromal cells they only reached an average speed of 0.200 ± 0.007 µm min^−1^ (Figure 8D). The measured speeds aligned closely with those reported in non‐printable thermogelated collagen type I hydrogels: approximately 0.5 µm min^−1^ for MDA‐MB‐231 cells in 1.2 mg mL^−1^ [16], 0.2 µm min^−1^ in 2 mg mL^−1^ [60], and 0.3 µm min^−1^ in 3 mg mL^−1^ collagen hydrogels [25]. The distribution plots for the mean speed and the speed of the fastest 10 % showed distinct shifts in the presence of ASCs, underscoring the impact of the ASCs on the tumor cell population (Figure 8C,D).

#### 2.7.3 Influence of ASCs on Cancer Cell Morphology and Collagen Structure

Time‐lapse analysis (Videos S2) revealed a shift in MDA‐MB‐231 cell morphology triggered by the presence of ASCs. Morphometric analysis confirmed this observation, showing that in the presence of ASCs, cancer cell sphericity was significantly reduced (Figure 9A). Cytoskeleton stainings demonstrated a predominantly elongated, spindle‐shaped phenotype of MDA‐MB‐231 cells in this condition, while in the absence of stromal cells, a substantial proportion of cancer cells displayed a more spherical morphology (Figure 9B). As morphological features have been proposed as potential predictors of migratory capacity in cancer cells [61], we aimed to elucidate the correlation between cell morphology and motility (Figure 9C–E and Figure S10). Migration speed increased steadily over approximately 40 h before stabilizing, while sphericity simultaneously decreased until it also reached a plateau. Notably, throughout the 95 h observation period, cancer cells in the presence of ASCs remained consistently faster and less spherical, i.e., more elongated than those cultured alone. Linear regression analysis of migration speed and sphericity over time revealed an inverse relationship both in the presence and absence of ASCs. The ASCs clearly influenced this correlation, which has not been shown previously. In the presence of ASCs, the negative slope of the regression line was distinctly steeper, indicating a stronger coupling between sphericity and speed (Figure 9E). An influence of stromal cells (cancer‐associated fibroblasts) on cancer cell morphology (SUM159) has been reported before; however, this was not associated with migration parameters [59].

Concomitant with the observed shift in tumor cell morphology, a distinct alteration in collagen structure was obvious over time (Figure 9F). In the acellular stroma condition, a collagen network with a rather homogeneous fiber structure characteristic of standard collagen hydrogels was apparent. In contrast, in the presence of ASCs, ECM remodeling toward a more condensed, irregular structure with thickened patches connected by thin fibers in interspaces could be observed. Properties of the collagen network can profoundly influence the migration of cancer cells in a 3D environment [1]. Similar to our findings, Liu et al. reported a more elongated protrusive phenotype and higher migration speed of MDA‐MB‐231 cells in a patchy, irregular collagen structure, which, in that case, however, was induced by mechanical agitation during in vitro fibrillogenesis of collagen [62]. In our study, the alteration of the collagen architecture was induced by stromal ASCs, which was likely an important factor contributing to the altered morphology and increased motility of the individual tumor cells in the presence of ASCs. ECM remodeling might trigger mechanotransduction pathways in the breast cancer cells via integrin signaling. This can drive the observed shift toward a more elongated, spindle‐shaped morphology mediated by downstream cytoskeleton regulators like Rho, Rac, and Cdc42 that translate structural matrix alterations into enhanced tumor cell invasiveness [63, 64]. A link between ASC‐mediated ECM remodeling and higher invasive capacity of premalignant breast cancer cells has recently been suggested based on observations in a spheroid‐based model for collective migration [7].

**FIGURE 9:**
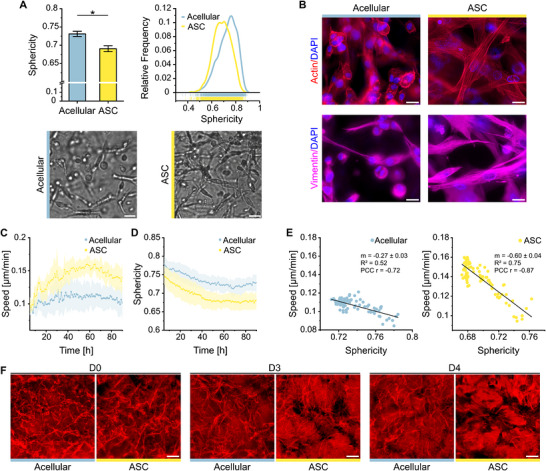
Analysis of MDA‐MB‐231 cell morphology, migration speed, and collagen matrix remodeling over time. (A) Mean track sphericity, corresponding distributions as density curves with rug plots indicating individual data points and brightfield images at *t* = 48 h of live‐cell imaging. Scale bar: 50 µm. Data are presented as mean of mean ± SE (n = 9). Significances were calculated using an unpaired Student´s *t*‐test. *p* < 0.05 is indicated by ^*^. (B) Cytoskeleton stainings of MDA‐MB‐231 cells in tumor‐acellular and in tumor‐ASC model after live‐cell imaging at day 5. Actin is depicted in red, vimentin in magenta, and DAPI in blue. Scale bar: 20 µm. (C) Speed at each time point given by the Euclidean distance between x/y/z‐positions of the same cell of consecutive frames divided by *Δt*  = 1 h, and (D) sphericity over time. Data are presented as mean ± SD. (E) Linear correlation between instantaneous speed and sphericity in tumor‐acellular and in tumor‐ASC model, respectively. (F) Collagen type I over time in the presence and absence of ASCs. Scale bar: 50 µm.

#### 2.7.4 Invasion of Breast Cancer Cells in Dependence on ASCs

Invasive breast cancer predominantly arises at the interface between fibroglandular and adipose tissue [65]. The invasion of tumor cells into adjacent adipose tissue represents a critical step in tumor dissemination [66, 67]. Therefore, breast cancer models that can replicate this process are imperative for future investigations of the underlying molecular mechanisms, such as the secretion of paracrine factors from neighboring cells. We recently demonstrated that paracrine secretion from ASCs and adipose spheroids altered MDA‐MB‐231 cancer cell migration in collagen type I hydrogels. However, in that model, stromal cells were embedded in a hyaluronic acid‐based bioink which prevented migration between compartments and thus precluded assessment of invasion [30].

Tumor organoids or spheroids, also as co‐spheroids with tumor cells and ASCs, have been widely used to investigate the invasion of tumor cells into the surrounding matrix; however, these models mimic collective tumor cell behavior rather than directed invasion of individual tumor cells into adjacent stromal tissue [7, 68, 69, 70, 71]. Invasion of individual breast cancer cells into an adjacent stromal compartment has been investigated in a recent study using a microfluidic cancer‐on‐chip model, and it was reported that stromal fibroblasts and macrophages had no influence on speed and invasion rate, but enhanced the directedness of cancer cell migration [58, 59]. However, adipose‐derived cells as part of the stromal compartment have not yet been included in such models.

Bioprinting offers distinct control over model geometry and a compartmentalized setup, and the two‐compartment model presented here enabled a comprehensive analysis of the modulatory effects of ASCs on cancer cell invasion dynamics. Our 3D model exhibits a native‐like collagen structure as an ECM stromal component. We investigated migration and invasion of tumor cells into the neighboring compartment, reflecting the invasion of single breast cancer cells into the adjacent stromal tissue as a key step in tumor dissemination. Importantly, although our model is analytically divided into two distinct compartments, the final hydrogel crosslinking is performed after printing both compartments, resulting in a unified hydrogel matrix without physical barriers impeding cellular invasion between compartments. Both in the presence and in the absence of ASCs, cancer cells invaded into the respective stromal compartment, as demonstrated in the time‐lapse recordings (Supplementary Videos S3) and corresponding z‐projections (Figure 10A).

**FIGURE 10:**
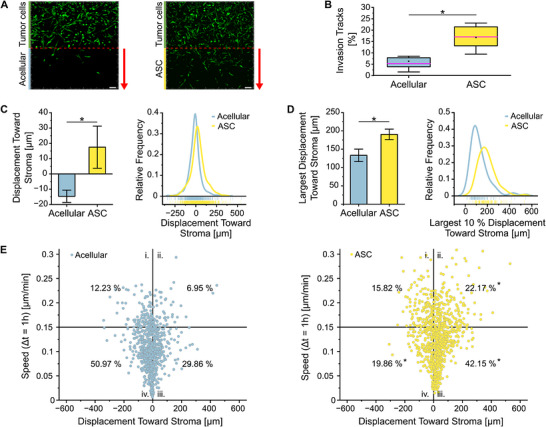
Analysis of cancer cell invasion into the stromal compartment. (A) Exemplary z‐projection of fluorescent MDA‐MB‐231 cancer cells in compartmentalized tumor‐stroma models at *t* = 95 h. The stromal compartment either contained ASCs or no cells (acellular). Considered cancer cell invasion is indicated by the red arrow. (B) Boxplot of invasion tracks: fraction of tracks that invaded into the stroma compartment at any point during the 95 h observation period. The box spans from the 25^th^ percentile to the 75^th^ percentile, representing the interquartile range (IQR). The horizontal line in magenta marks the median, while the black dot represents the mean. The whiskers extend to the minimum and maximum values within 1.5 times the IQR. (C) Displacement toward the stroma compartment (*Y_Displacement_* = *Y_max_* − *Y*_*t* = 0_, whereby *Y*_*t* = 0_ > 300 µm) Left: average displacement toward stroma. Right: distribution of displacements toward stroma as density curves with a rug plot indicating individual data points. (D) Largest 10 % displacement toward the stroma compartment. (C, D) Data are presented as mean of mean ± SE (n = 9). Significances were calculated using an unpaired Student's *t*‐test. *p* < 0.05 is indicated by *. (E) Scatter plots of single‐cell migration speed versus displacement toward stroma in the tumor‐acellular and tumor‐ASC model. Each point represents an individual track. Vertical and horizontal lines indicate zero displacement and the median migration speed, respectively, dividing the plots into four quadrants. Percentages represent the proportion of cells within each quadrant. Quadrant distributions differ significantly between the two conditions (χ^2^(3) = 200.10, *p* < 0.0001). Post‐hoc analysis was performed between corresponding quadrants of both conditions (quadrant i vs. i, quadrant ii vs. ii, etc.) using Bonferroni‐corrected pairwise comparisons (α = 0.00625); quadrants that differ significantly from their counterpart in the other condition are indicated by ^*^.

Quantitative analysis showed that, over time, a significantly higher fraction of cancer cells migrated into the ASC‐laden compartment (17.4 ± 1.6 %) compared to the acellular compartment (7.0 ± 1.3 %) (Figure 10B). Moreover, our setup enabled detailed characterization of directed migration toward the stromal compartment via directed trajectory analysis in the Y‐direction (difference between the maximal Y‐position and the initial Y‐position of each track), thereby capturing the extent to which tumor cells migrated toward and into the stroma. To ensure accurate measurement of bidirectional movement, both toward and away from the stromal compartment, we focused our analysis on cancer cells that were located at least 300 µm from the edge of the ROI at the start of imaging, as illustrated in Figure S11. In the presence of ASCs, cancer cells exhibited significantly greater displacements toward the stromal compartment (17.50 ± 13.8 µm), whereas in the absence of stromal cells, cancer cells migrated on average in the opposite direction, with a displacement of −14.66 ± 4.05 µm (Figure 10C). When considering only the top 10 % of cells exhibiting the largest displacement toward stroma (i.e., positive Y‐direction migration), cells in the ASC‐laden condition still covered a significantly greater displacement (190 ± 14 µm) compared to the ASC‐free condition (133 ± 17 µm) (Figure 10D). These results indicate that ASCs play a key role in directing cancer cell migration. This may be, at least partly, due to chemotactic factors secreted by ASCs such as CXCL12 and CCL5, which are described to generally promote tumor cell migration and invasion [72, 73]. Directional migration toward an adipose compartment was also recently reported for MCF‐7 breast cancer cells; however, as migration was not the primary focus, that study relied on endpoint analysis of spheroid invasion areas without capturing single invasion dynamics [74].

To better understand the dynamics of individual cell motility within the tumor cell population and to what extent this is influenced by the stromal cells, we plotted the speed of individual cell tracks versus their directionality, i.e., the displacement toward stroma, for both conditions (acellular vs. ASC‐laden stromal compartment) (Figure 10E). In the scatter plot of the ASC‐free condition, limited heterogeneity in the migration behavior of the tumor cells was apparent, with only a small proportion of cells migrating rapidly and in a directed manner. The majority of tracks clustered around low speeds with minimal or negative stromal displacement, indicating predominance of slow, non‐stromal directed movements. In contrast, the presence of ASCs induced a distinctly higher heterogeneity in the migration behavior of the tumor cells, with a significant increase in the fraction of fast, stromal‐directed cells (quadrant ii; 22.2 % vs. 6.9 %) (Figure 10E). This shift indicated that ASCs did not uniformly enhance the motility of the tumor cell population; rather, they specifically boosted a subset of tumor cells that were characterized by fast movement toward or into the adjacent stroma. To our knowledge, this is the first study to demonstrate such an effect at a single‐cell level, reflecting the heterogeneity of tumor cells in their migratory response to biochemical and/or mechanical cues provided by ASCs. Such heterogeneity can result in varying sensitivity to drugs within the tumor cell population and, for example, reduce or prevent the efficacy of targeted therapeutics designed to prevent tumor cell dissemination [75, 76].

Thus, characterizing such highly motile and invasive subsets of breast cancer cells promoted by adipose stromal cells could advance the identification of molecular subpopulations with distinct migratory behaviors in subsequent studies and offer critical insights into the mechanisms that drive differential invasion dynamics. This may facilitate the development of more specific and effective therapeutic options to prevent or decelerate tumor cell dissemination. Moreover, the efficacy of existing therapeutics could be evaluated more precisely regarding their ability to specifically target these highly motile and invasive cells.

## 3 Conclusion

This study presents a fully 3D bioprinted breast cancer model that specifically enables spatiotemporal analysis of individual tumor cell migration and invasion at the tumor‐stroma interface. As a prerequisite, a bioink that is both migration‐permissive and printable was developed based on low‐concentration functionalized collagen type I and hyaluronic acid, providing high print fidelity and structural integrity. The general suitability of the bioink for migration studies was shown using spheroid invasion assays. In the bioprinted compartmentalized co‐culture model, real‐time tracking of migrating tumor cells revealed that ASCs located in the stromal compartment strongly promoted tumor cell migration and invasion dynamics, characterized by increased migration speed, migration distance, and invasion into stroma. This was accompanied by a remodeling of the collagen matrix and a shift in tumor cell morphology toward a spindle‐shaped protrusive phenotype in the presence of ASCs. A distinct correlation between cell morphology and migration speed was found, which was strongly modulated by ASCs. Detailed analysis of the directed movement of individual tumor cells highlighted the heterogeneity of the tumor cells in terms of their migratory behavior and identified a subset of tumor cells with high speed and high directionality toward the stromal compartment, which was significantly enhanced by the presence of ASCs.

Detailed molecular profiling of individual tumor cells with pronounced migratory and invasive phenotypes triggered by stromal cells may hold promise for the discovery of new biomarkers and the development of targeted therapies aimed at preventing tumor dissemination. As the modular configuration of the printed model supports increasing complexity, additional stromal cell types, such as immune cells, can be easily integrated in future studies. The model could also be extended to include additional ECM proteins to further mimic the complexity of the TME. Overall, this innovative approach represents a substantial advancement in functional tumor modeling and provides an adaptable framework for investigating tumor‐stroma dynamics, ultimately paving the way for the development of targeted therapeutic strategies against invasive cancer.

## 4 Experimental Section/Methods

### 4.1 Preparation of ColMA/HA‐SH Bioink

Lyophilized ColMA (Advanced Biomatrix, Carlsbad, CA, USA) was reconstituted in acetic acid (20 mm) over a period of two days at 4 °C while shaking, yielding stock solutions of 6 or 8 mg mL^−1^, which were stored at 4 °C until use. Prior to combining it with the other hydrogel components, ColMA was neutralized using NaOH. For all experiments, thiol‐modified hyaluronic acid (HA‐SH; 498 kDa, DS: 50 %), synthesized as previously described [77], and lithium‐phenyl‐2,4,6‐trimethylbenzoylphosphinate (LAP; Sigma‐Aldrich, St. Louis, MO, USA) were freshly prepared. Both chemicals were dissolved in HEPES buffer (154 mm, pH = 7.6) to obtain a final concentration of 1 and 0.8 mg mL^−1^, respectively, and added to the ColMA stock solution (final concentration of ColMA varied between 1.5 – 5.5 mg mL^−1^ depending on the formulation). Subsequently, the hydrogel precursor solution was incubated on ice, protected from light, for 45 min. Depending on the experiment, the ink was printed or cast before final crosslinking via UV radiation (405 nm, 4.4 W cm^−2^ for 2 min).

### 4.2 3D Printing and Bioprinting

3D printing was performed with a BioX 3D bioprinter (Cellink, Stockholm, Sweden) at 4 °C using 3 cc printing cartridges (Nordson EFD, Westlake, OH, USA) and 23 G (330 µm) blunt steel nozzles (Nordson EFD) unless stated otherwise. For bioprinting, the hydrogel precursor solution was mixed with MDA‐MB‐231, GFP‐expressing MDA‐MB‐231, or ASCs (2 × 10^6^ cells mL^−1^), transferred to the printing cartridges, incubated for 45 min on ice, and printed at an applied pressure of 30 – 40 kPa. All G‐codes were generated with HeartWare 2.4.1 (Cellink, Boston, MA, USA). Printability of different bioink formulations (1/1.5/2.5/3.5/4.5/5.5 mg mL^−1^ ColMA + 1 mg mL^−1^ HA‐SH + 0.8 mg mL^−1^ LAP) was assessed using three complementary tests: grid structure test, filament fusion test, and 4‐angled pattern test [77, 78]. For the grid structure test, square‐shaped pores were printed in a 5 × 5 pattern yielding a 15 mm^2^ construct. For the filament fusion test, 17 parallel strands were printed with inter‐strand distances decreasing progressively from 2 mm to 0.5 mm, thus illustrating the minimum inter‐strand distance preventing filament merging. For the 4‐angled pattern test, a continuous 23 mm line including turns of 125°, 90°, 55°, and 20° was printed. All constructs were imaged post‐printing using an OZL‐464 stereo zoom microscope (KERN, Balingen, Germany) and analyzed using Olympus cellSens Dimension Imaging Software (version 4.2; Evident Scientific, Tokyo, Japan) and ImageJ (v 1.54f) to obtain the following resolution parameters. The strand intersection diagonal, defined as the diagonal of two crossing filaments, was measured from the grid structure test. The filament width was determined from both the filament fusion test and from the second linear segment of the 4‐angled pattern test. From this segment, the uniformity ratio and the turn angular error were evaluated at the 125° and 90° turns as the absolute difference between the designed and printed angle, as described by Gillispie et al. [78].

### 4.3 Rheological Characterization

Rheological properties of ColMA/HA‐SH formulations were characterized using an Anton Paar MCR 702 rheometer (Anton Paar, Graz, Austria) equipped with a 25 mm parallel plate geometry (gap: 500 µm) and a solvent trap to limit solvent evaporation during measurements. For all experiments, the hydrogel precursor solution was prepared fresh and subsequently loaded on the lower plate at room temperature. To determine the viscosity profile of the formulations, shear rate sweep measurements were performed from 1 to 100 s^−1^ on the liquid precursor solutions before photocrosslinking. The time sweep measurements were conducted with 0.5 % shear rate and an angular frequency of 10 rad s^−1^, extracted from the linear viscoelastic region. To induce photocrosslinking, a UV–vis light source including an optical filter (390 – 500 nm; bluepoint 4, Dr. Hönle AG, Gilching, Germany) and a flexible light guide were added to the rheometer. Post photocrosslinking, frequency sweeps from 100 to 1 rad s^−^^1^ at a shear strain of 1 % were conducted.

### 4.4 Mechanical Characterization

Cyclic compression tests of ColMA/HA‐SH constructs at day 1 with three cycles, a maximum strain of 40 %, and a velocity of 40 µm s^−1^ were performed using a Discovery HR‐3 rheometer from TA instruments (New Castle, DE, USA) equipped with an 8 mm plate‐plate geometry. We assume that the hydrogels slide along the specimen holders′ surfaces, resulting in a homogeneous deformation throughout the hydrogels. To ensure hydration and to mimic culture conditions during testing, samples were hydrated with some drops of cell culture media during testing, and all tests were performed at 37 °C. Images of each sample were taken right before testing, and the diameter of each sample was determined using Fiji/ImageJ (v 1.54f) [79]. Subsequent hyperelastic modeling using the modified one‐term Ogden model strain‐energy function was used to determine the apparent Young's modulus (see Supporting Information for details of analysis).

### 4.5 Analysis of Ultrastructure of ColMA/HA‐SH Hydrogels

To observe the ultrastructure of ColMA/HA‐SH constructs, samples were assessed via confocal reflection microscopy and scanning electron microscopy. For the latter, printed or cast ColMA/HA‐SH hydrogels (3.5 mg mL^−1^ ColMA + 1 mg mL^−1^ HA‐SH + 0.8 mg mL^−1^ LAP) were washed with ice‐cold PBS, fixed with 6 % glutaraldehyde (Carl Roth, Karlsruhe, Germany) on ice prior to dehydration via ascending acetone series (30 to 100 %) at room temperature. ASC‐laden ColMA/HA‐SH hydrogels were prepared at a cell density of 2 × 10^6^ cells mL^−1^. All samples were subsequently critical point dried (Critical Point Dryer CPD 030, Bal‐Tec, Witten, Germany), mounted on stubs using carbon adhesive discs and sputtered with a 4 nm layer of platinum in a sputter coater (Leica Microsystems ACE 400, Wetzlar, Germany) prior to imaging using a Crossbeam 340 field emission scanning electron microscope (Zeiss, Oberkochen, Germany) at an acceleration voltage of 2 kV. Fiber diameters were determined using Olympus cellSens Dimension 4.1 imaging software by measuring 50 fibers of n = 3 independent images. To visualize collagen fibers of different formulations (2.5/3.5/5.5 mg mL^−1 ^ColMA + 1 mg mL^−1^ HA‐SH + 0.8 mg mL^−1^ LAP and 2.5 mg mL^−1^ ColMA + 0.8 mg mL^−1^ LAP) without dehydrating the hydrogels, we used an upright laser scanning confocal microscope (SP5, Leica) in confocal reflection mode equipped with a Leica 20x dip‐in water‐immersion objective (HCX APO L, Leica) and acquired an image volume of 180 µm^3^ with a voxel size of 0.36 × 0.36 × 0.59 µm. For 3D reconstructions of confocal reflection microscopy stacks, we used the Python package SAENOPY [80]. The pore size diameter of the hydrogels was determined as described previously in Lang et al. [81] using the upper 65 µm of the image stacks.

### 4.6 Cell Culture

MDA‐MB‐231 breast cancer cells (ATCC, Manassas, VA, USA) and human ASCs (Lonza, Basel, Switzerland) were cultured in Dulbecco's Modified Eagle's Medium/Ham's F‐12 (Thermo Fisher, Waltham, MA, USA) supplemented with 10 % fetal bovine serum (Thermo Fisher), 1 % penicillin/streptomycin (100 U mL^−1^ penicillin, 0.1 mg mL^−1^ streptomycin; Thermo Fisher) at 37 °C and 5 % CO_2_ until 80 – 85 % confluency. ASCs were additionally supplemented with basic fibroblast growth factor (3 ng mL^−1^; BioLegend, London, UK) at 37 °C and 5 % CO_2_. The media was changed every other day. Cells (passage 5 at 80 – 85 % confluency) were harvested using trypsin‐EDTA solution (0.25 %; Thermo Fisher) or adipogenic differentiation was initiated. For the latter, cell expansion media was additionally supplemented with insulin (1.7 × 10^−6^ m; PromoCell, Heidelberg, Germany), dexamethasone (1 × 10^−6^ m; Sigma‐Aldrich), 3‐isobutyl‐1‐methylxanthine (IBMX, 5 × 10^−4^ m; Serva Electrophoresis, Heidelberg, Germany), and indomethacin (2 × 10^−4^ m; Sigma‐Aldrich).

### 4.7 Analysis of Cell Viability

Cell viability within hydrogel constructs was assessed using a live/dead cell staining kit (PromoKine, Heidelberg, Germany). Samples were washed with PBS, incubated in live/dead staining solution (4 × 10^−6^ m ethidium homodimer III, 1 × 10^−6^ m calcein acetoxymethyl ester) for 45 min at room temperature, on an orbital shaker, and imaged using an inverted fluorescence microscope (Olympus IX51/XC30). For quantification, respective extended focus images were analyzed using ImageJ by initially transforming them into 8‐bit format, after which the Yen threshold was implemented, and the cells were quantified utilizing the analyze particle tool.

### 4.8 Spheroid Generation and Spheroid Invasion Assay

For the generation of MDA‐MB‐231 and MDA‐MB‐231‐GFP spheroids (2´500 cells/spheroid), 3D Petri Dishes (MICROTISSUES technology, MicroTissues Inc., Providence, RI, USA) were used according to the manufacturer's instructions. To enhance spheroid formation, cell‐laden micro‐molds were settled for 15 min in the incubator (37 °C, 5 % CO_2_) before centrifugation for 10 min at 1´000 g and the addition of cell culture media (2 mL per well). After 24 h, cell‐laden micro‐molds were centrifuged for 10 min at 500 g. Spheroids were harvested at day 2. Depending on the experiment, hydrogel precursor solutions consisting of 2.5 mg mL^−1^ or 3.5 mg mL^−1^ ColMA, 1 mg mL^−1^ HA‐SH, and 0.8 mg mL^−1^ LAP were incubated for 45 min on ice and subsequently cast into customized silicon molds (diameter: 5 mm, depth: 2 mm) using a positive displacement pipette. A single MDA‐MB‐231 cell spheroid was placed in the center of the hydrogel prior to final UV‐induced crosslinking. Constructs were cultured in 24‐well plates with cell culture media or cell culture media supplemented with the ROCK inhibitor Y27632 (30 µM) and/or the MMP inhibitor Batimastat (10 µM) (both from Tocris, Bristol, United Kingdom) at 37 °C and 5 % CO_2_. To observe cell migration into the surrounding matrix over time, phase contrast images were taken at different time points with the inverted microscope Olympus IX51. For quantification, a custom‐written Fiji script was used to determine the area occupied by the spheroid through image segmentation. Briefly, to correct for uneven backgrounds, the “subtract background” function was applied to the images, generating a background that was subsequently subtracted from the respective original images. Corrected images were converted to binary masks, and to enhance spheroid segmentation, a series of operations — fill holes, dilate, and erode — were applied. Finally, the spheroid area was detected using the “measure” command. For the spheroid tumor‐stroma model, ASCs or adipocytes or no cells were suspended in hydrogel precursor solution consisting of 2.5 mg mL^−1^ ColMA, 1 mg mL^−1^ HA‐SH, and 0.8 mg mL^−1^ LAP followed by a 45 min incubation on ice and subsequent single spheroid placement as described above. For quantification, only the fluorescent channel was used for image segmentation with a custom Fiji script. Briefly, for background correction, we measured the mean fluorescence of the background and subsequently subtracted it from the entire original image. The auto threshold “triangle” was applied to the corrected images, and to enhance spheroid segmentation, a series of operations — fill holes, dilate, and erode — were applied. Finally, the spheroid area was detected using the “measure” command.

### 4.9 Compartmentalized Tumor‐Stroma Model

To study in detail the migration and invasion of MDA‐MB‐231 cells in response to neighboring ASCs, we employed a compartmentalized, fully 3D bioprinted tumor‐stroma model. The model consisted of two distinct compartments, both printed with ColMA/HA‐SH bioink: a tumor compartment containing MDA‐MB‐231 cells, which stably expressed cytosolic GFP, and a stromal compartment containing either ASCs (ASC compartment, labeled “ASC” in the respective figures) or no cells (acellular compartment, labeled “acellular” in the respective figures). Cells were embedded in 3.5 mg mL^−1^ ColMA + 1 mg mL^−1^ HA‐SH + 0.8 mg mL^−1^ LAP at a cell density of 2 × 10^6^ cells mL^−1^. Bioink preparation and bioprinting were performed as described above (4 °C, 30 – 40 kPa, 330 µm blunt steel nozzle). The tumor‐stroma models measured 8 × 2.7 mm in total dimensions (see G‐code in Figure 7A). The constructs were symmetrically divided into tumor and stroma compartments, each consisting of five parallel strands with equal width and spacing (0.5 mm). To facilitate imaging, the constructs were printed into removable silicon chambers (IBIDI, Gräfelfing, Germany), which were attached to cover slips (1.5H, 24 × 60 mm). Following UV crosslinking, samples were incubated for 12 h in the incubator (37 °C, 5 % CO_2_) before being mounted on a custom 3D‐printed sample holder for live‐cell imaging. Time‐lapse fluorescence and brightfield images were acquired using a motorized microscope (DMI6000 Leica) equipped with a temperature and CO_2_‐controlled incubator (37 °C, 5 % CO_2_), 10x magnification objective (HC FL PLAN 10x/0.25 DRY; Leica), and a CMOS camera (Hamamatsu‐Flash4‐USB3‐002889; Hamamatsu Photonics K.K., Hamamatsu City, Japan). Image stacks with a height of ∼350 µm up to 1 mm, depending upon the sample height, were captured at 1 h intervals over a period of 95 h at 3 different fields of view per sample covering the interface of the two different compartments. Images were acquired with a voxel size of 0.65 × 0.65 × 6.1 µm and the fluorescence channel was deconvolved using the express deconvolution option in Huygens Professional 24.10 (SVI, Hilversum, Netherlands). Segmentation and tracking analyses of the GFP‐expressing MDA‐MB‐231 cells were performed using Imaris 10.1 (Oxford Instruments, Abingdon, UK). For segmentation, we used a surface grain size of 1.3 µm and applied a threshold value of 2, and excluded all objects below 200 voxels. Cells were tracked using the autoregressive motion model with a maximal distance of 45 µm, a maximal gap size of 3, and enabled ‘fill gaps’ option. To calculate the motile fraction of cells, cells whose track length was > 20 µm were considered motile. Only tracks with a track length > 20 µm and a continuous track duration > 24 h were considered for the following analysis (n = 9, whereby each n comprised ∼450 tracks): (i) track length defined as the total length of displacements within the track, i.e, Euclidean distance between x/y/z‐positions ($L=\sum_{t=t_{F}+1}^{t_{L}}\sqrt{D_{x}{\left(t,t-1\right)}^{2}+D_{y}{\left(t,t-1\right)}^{2}+D_{z}{\left(t,t-1\right)}^{2}}\;$ whereby *L* = track length, *t_L_* = last time index of track, *t_F _* = first time index of track, and the displacement of the object is defined as *D*_*x*; *y*; *z*_(*t*_1_,*t*_2_) = *P*_*x*; *y*; *z*_(*t*_1_) − *P*_*x*; *y*; *z*_(*t*_2_) whereby *P_x;y;z_* is the x, y, or z position of the object at the time index *t*), (ii) track duration (difference between the initial and the final time point of the track), and (iii) migration speed over time (given by $S\;\left(t\right)=\frac{\sqrt{D_{x}{\left(t,t-1\right)}^{2}+D_{y}{\left(t,t-1\right)}^{2}+D_{z}{\left(t,t-1\right)}^{2}}}{\Delta t}$, whereby *Δt = 1 h*). Parameters were averaged over all cells within an ROI. The average speed was calculated by averaging the migration speed between consecutive frames over the observation period for each cell trajectory and then over all cells within an ROI. Sphericity at each time point was determined by the ratio of the surface area of a sphere of the same volume as the cell *V_c_* to the actual surface area *A_c_* of the cell, $\psi =\frac{\pi^{\frac{1}{3}}\;{\left(6V_{c}\right)}^{\frac{2}{3}}}{A_{c}}$ [82] and mean track sphericity was calculated by first averaging sphericity over time for each cell and then computing the mean across all tracks within an ROI. To quantify cell displacement toward the stromal compartment (Y‐displacement), we computed the difference between the maximum Y position reached by each cell and its initial Y coordinate at *t = 0* (*Y_Displacement_* = *Y_max_* − *Y*_*t* = 0_). Only cells with an initial Y position greater than 300 µm were included in the analysis, ensuring that each cell's movement could be fully tracked in both the negative and positive Y directions (see Figure S11 for illustration). For each trajectory, the correlation between Y‐displacement and average track speed was evaluated. Data were visualized using scatter plots, and relative frequencies of trajectories in each quadrant were calculated to assess directional trends. For maximal track length, maximal displacement toward stroma, and maximal speed, only the top 10 % values of the respective parameters within an ROI were considered. To determine the invasion tracks, the percentage of tracks that appeared in the stromal compartment with respect to the total number of tracks was calculated.

### 4.10 Immunostaining

Printed ColMA/HA‐SH constructs were washed with PBS and fixed in 4 % buffered formaldehyde (1 h, RT). Samples were then washed in PBS containing 1 % bovine serum albumin and 0.2 % TritonX‐100 (both from Sigma Aldrich) three times. For combined antigen retrieval and blocking, samples were incubated in the same solution for 1 h at RT. After washing with PBS (3 × 10 min), constructs were incubated with one of the following staining solutions: ActinRed 555 ReadyProbe (2 drops/mL in PBS; Thermo Fisher), anti‐vimentin(1:200; ab92547, Abcam, Cambridge, UK) in antibody diluent (Agilent Dako, Santa Clara, CA, USA) overnight at RT, anti‐collagen type I (1:300; MA1‐26771, Thermo Fisher) in antibody diluent 7 h at RT. After primary staining, actin‐ and vimentin‐stained samples were counterstained with DAPI solution (1 µg mL^−1^ in PBS; Thermo Fisher) for 15 min at RT. All samples were imaged using an inverted fluorescence microscope (Olympus IX51).

### 4.11 Statistical Analysis

Statistical analysis was performed using OriginPro2023 10.0.0.154. Comparisons between two groups were conducted via an unpaired two‐sample Student's *t*‐test or Welch's *t*‐test. For multiple comparisons, we used one‐way or two‐way ANOVA followed by Tukey–Kramer post hoc test. *P*‐values < 0.05 were considered statistically significant and are indicated by *. Results are expressed as the mean ± standard error (SE) or mean ± standard deviation (SD). A chi‐square test of independence was conducted to test for associations between categorical data (α = 0.05). When the overall test was significant, pairwise post‐hoc comparisons were performed using Bonferroni correction.

## Funding

The project was funded by the Deutsche Forschungsgemeinschaft (DFG, German Research Foundation) – Project number 326 998 133 – TRR 225 (subprojects C02, A01, A02, B06, B09, Z02).

## Conflicts of Interest

The authors declare no conflict of interest.

## Supporting information




**Supporting File 1**: adhm71353‐sup‐0001‐SuppMat.pdf.

**Supporting File 2**: adhm71353‐sup‐0002‐VideoS1.mp4.

**Supporting File 3**: adhm71353‐sup‐0003‐VideoS2.mp4.

**Supporting File 4**: adhm71353‐sup‐0004‐VideoS3.mp4.

## Data Availability

The data that support the findings of this study are available from the corresponding author upon reasonable request.
