# Supplementary material for: 3D Bioprinted Breast Cancer‐Stroma Model with Tailored Migration‐Permissive Bioink Reveals Impact of Adipose‐Derived Stromal Cells on Cancer Cell Migration and Invasion Dynamics
Source: Adv Healthc Mater. 2026 Jul 26;15(30):e71353. doi: 10.1002/adhm.71353 (PMC13474114; doi:10.1002/adhm.71353)
Supplement: Supplementary file 1 [file ADHM-15-0-s003.pdf]

Supporting Information

3D Bioprinted Breast Cancer-Stroma Model with Tailored Migration-Permissive Bioink Reveals Impact of Adipose-Derived Stromal Cells on Cancer Cell Migration and Invasion Dynamics

Sabrina Stecher, Joachim Schenk, Alessandro Cianciosi, Katherina Hemmen, David Böhringer, Franziska Dusi, Jessica Faber, Philipp Stahlhut, Benedikt Gantert, Emilie Rouenhoff, Annika Gmöhling, Tessa Lühmann, Jörg Teßmar, Jürgen Groll, Silvia Budday, Tomasz Jüngst, Ben Fabry, Katrin G. Heinze, Petra Bauer-Kreisel, Torsten Blunk*

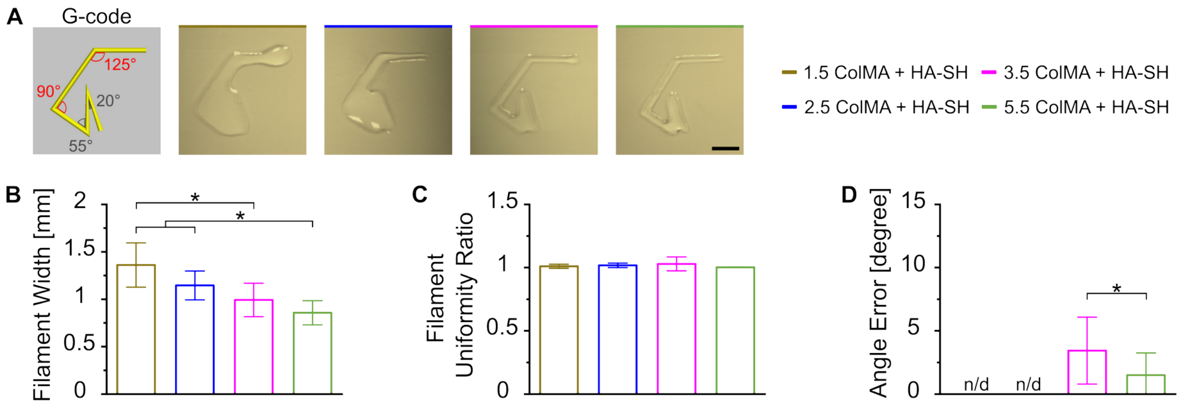

Figure S1. Printability assessment of different bioink formulations using the 4-angled pattern test. (A) G-code design and representative stereomicroscopy images of printed constructs for each bioink formulation. The test pattern comprised a continuous 23 mm line incorporating turns of 125°, 90°, 55°, and 20°. Scale bar: 3 mm. (B) Filament width and (C) filament uniformity ratio were quantified from the second linear segment, located between the 125° and 90° turns. (D) Quantification of the turn angle error, defined as the absolute difference between the designed and the printed angle, at the 125° and 90° turns. Data are presented as mean ± SD (n = 6-10 for filament width and uniformity ratio; n = 10-20 for angle error). Statistical significance was determined by one-way ANOVA followed by Tukey-Kramer post hoc test (B,C) or unpaired Student's t-test (D; n/d: not determined); * $p < 0.05$.

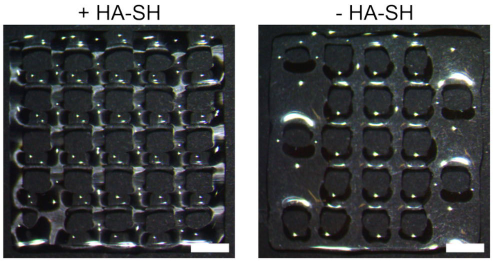

Figure S2. Grid Structure test of bioinks with 2.5 mg mL-1 ColMA and either 1 mg mL-1 HA-SH or no HA-SH showing that the addition of HA-SH enhances the printability of the bioink. Scale bar: 3 mm.

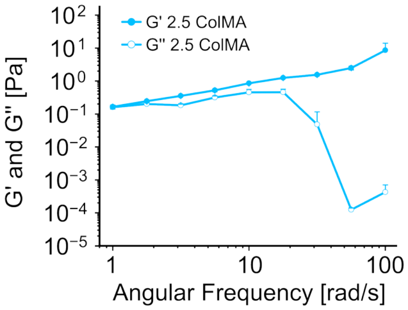

Figure S3. Frequency sweeps of bioink with 2.5 mg mL-1 ColMA (no HA-SH) after photocrosslinking. Data are presented as mean ± SD (n = 3).

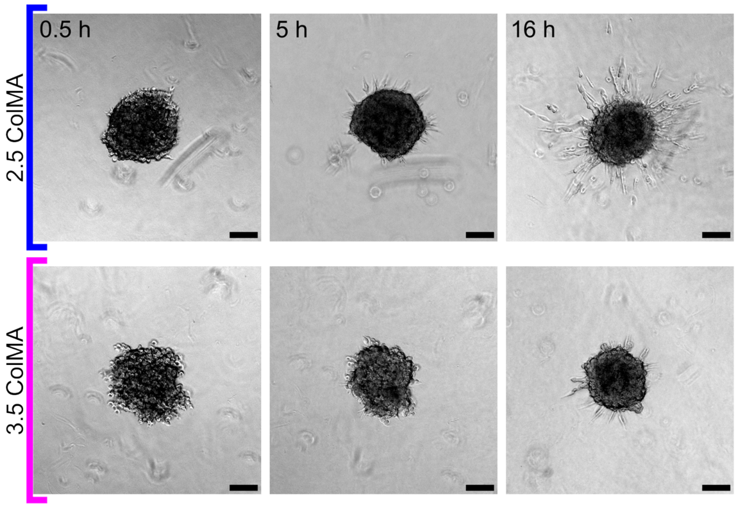

Figure S4. Phase contrast images of MDA-MB-231 spheroids embedded in 2.5 mg ml^{-1} or 3.5 mg ml^{-1} ColMA/HA-SH bioink over time. Scale bar: 100 µm.

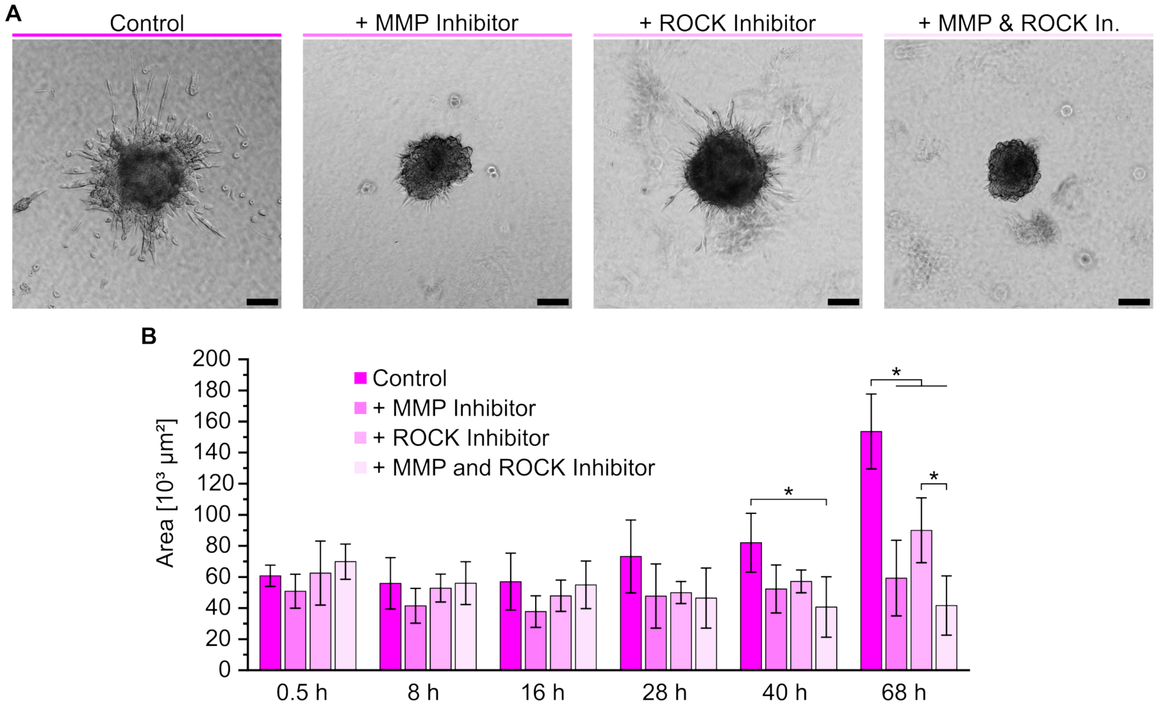

Figure S5. Analysis of the mode of cell migration of MDA-MB-231 spheroids in 3.5 mg mL^{-1} ColMA/HA-SH hydrogels cultured in cell culture media or cell culture media supplemented with MMP inhibitor, ROCK inhibitor or both. (A) Representative phase contrast images of MDA-MB-231 spheroids in ColMA/HA-SH hydrogels after 68 h in culture. Scale bar: 100 µm. (B) Quantification of spheroid invasion assay depicting the spheroid area over time. Data are presented as mean ± SD (n = 3-5). Significances were calculated using one-way ANOVA followed by Tukey-Kramer post hoc test ($p < 0.05$ is indicated by *).

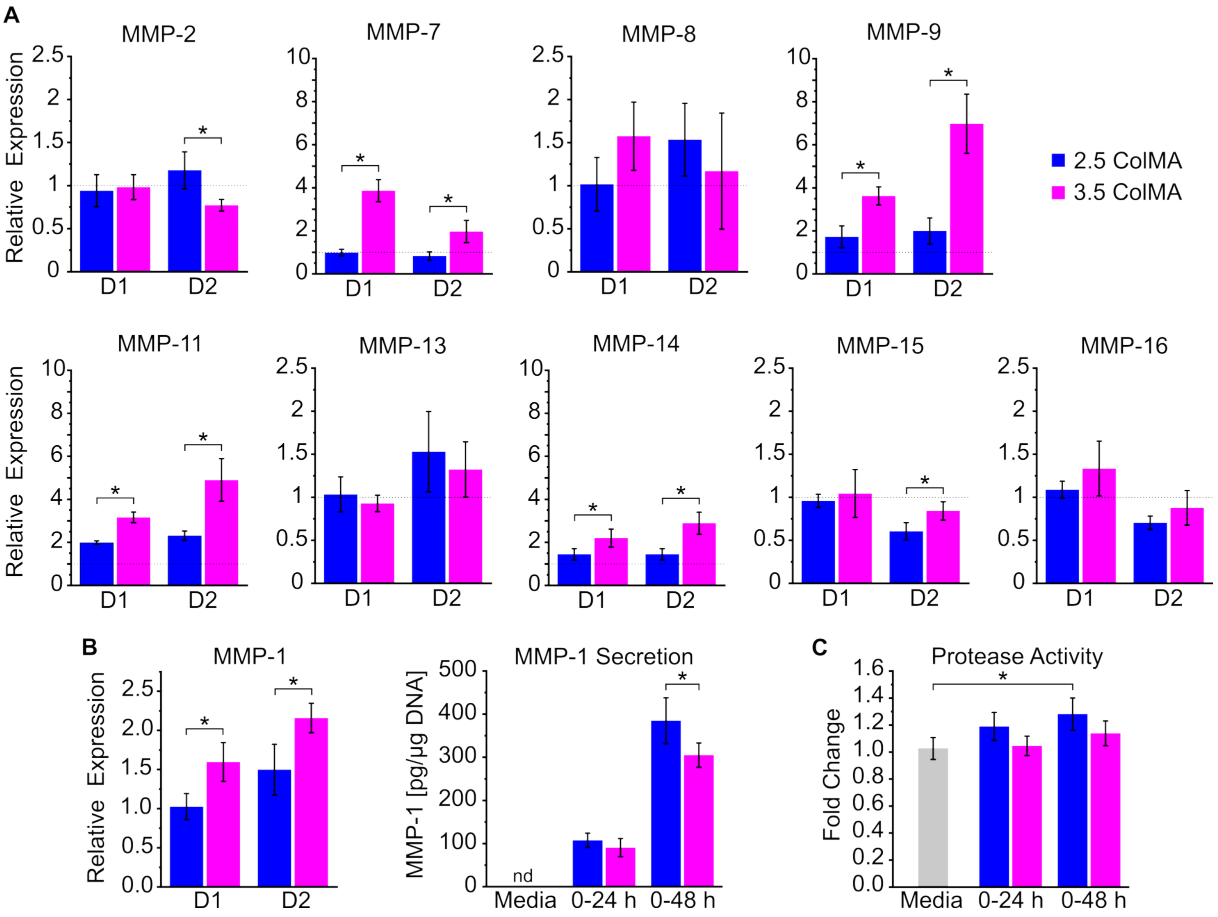

Figure S6. Analysis of gene expression and proteolytic activity of matrix metalloproteases of MDA-MB-231 cells embedded in 2.5 mg mL^{-1} or 3.5 mg mL^{-1} ColMA/HA-SH hydrogels. (A) MMP-2, MMP-7, MMP-8, MMP-9, MMP-11, MMP-13, MMP-14, MMP-15, and MMP-16 gene expression determined by RT–qPCR at day 1 and day 2 (n = 4). Gene expression was normalized to EF1α; obtained values were further normalized to day 0 of 2.5 mg mL^{-1} ColMA/HA-SH samples (dashed line). (B) Analysis of MMP-1 expressed by MDA-MB-231 cells embedded in ColMA/HA-SH hydrogels. Gene expression determined by RT–qPCR at day 1 and day 2 was normalized to EF1α; obtained values were further normalized to day 0 of 2.5 mg mL^{-1} ColMA/HA-SH samples. MMP-1 protein expression levels determined via ELISA after 24 h and 48 h were normalized to the DNA of the respective construct (n = 4). (C) Fold change of general protease activity of MDA-MB-231 cells embedded in ColMA/HA-SH hydrogels normalized to media + FCS (reporter peptide: GPAG / LRGG; n = 3). All data are shown as mean ± SD. Significances were calculated using one-way ANOVA followed by Tukey-Kramer´s post hoc test ($p < 0.05$ is indicated by *).

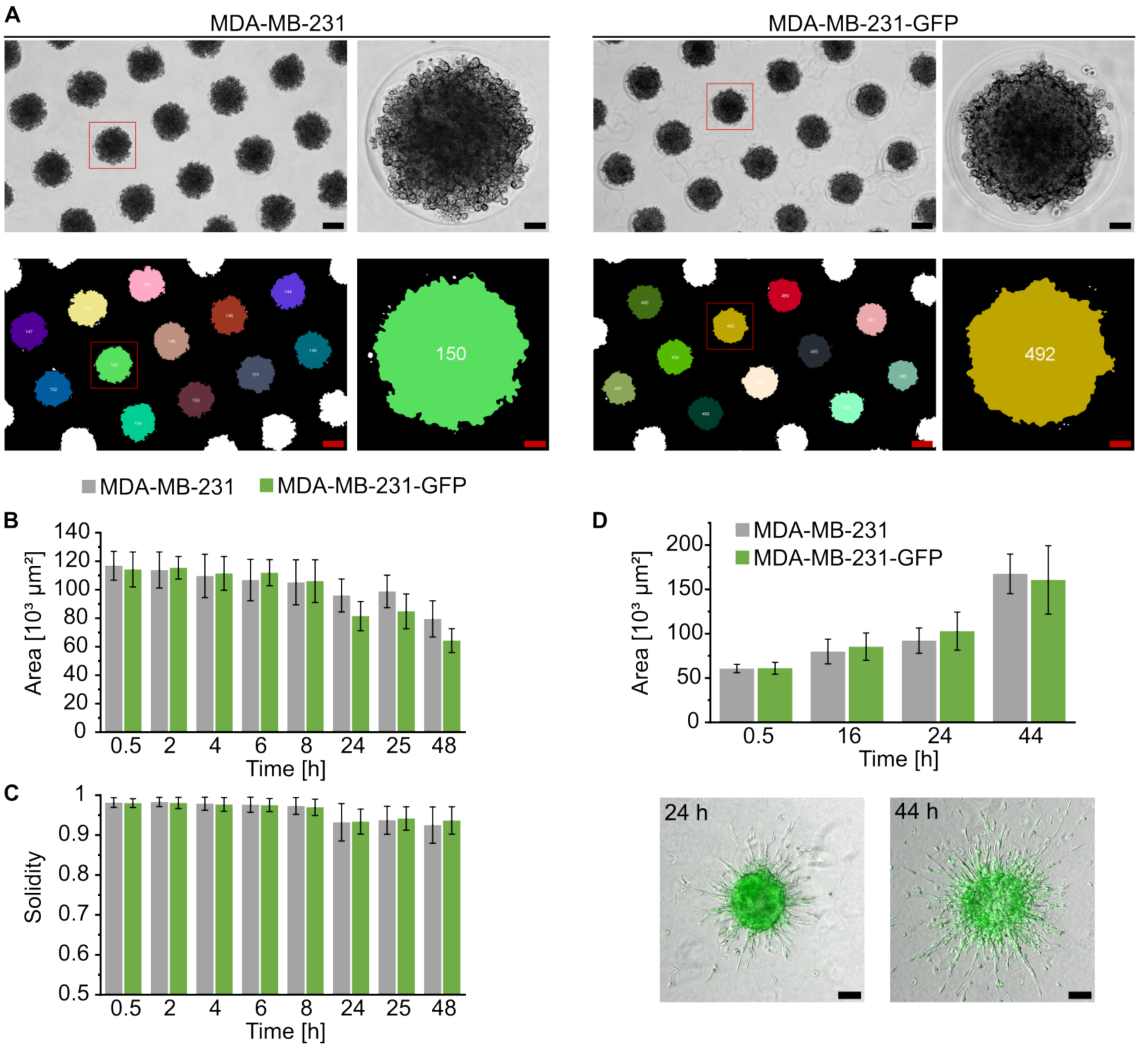

Figure S7. Comparison of GFP and non-GFP expressing MDA-MB-231 spheroids. (A) Phase contrast images of MDA-MB-231 or GFP expressing MDA-MB-231-GFP spheroids (2´500 cells/spheroid) in agarose micromolds and the respective segmentation masks which were used for analysis. Scale bar: 200 µm and 50 µm. (B) Area and (C) solidity of MDA-MB-231 and MDA-MB-231-GFP spheroids over time (n = 4-5 micromolds, 20-25 spheroids per micromold). (D) MDA-MB-231 and MDA-MB-231-GFP spheroid invasion assay in 2.5 mg mL^{-1} ColMA/HA-SH hydrogel (n = 3-7). Scale bar: 100 µm. All data are shown as mean ± SD.

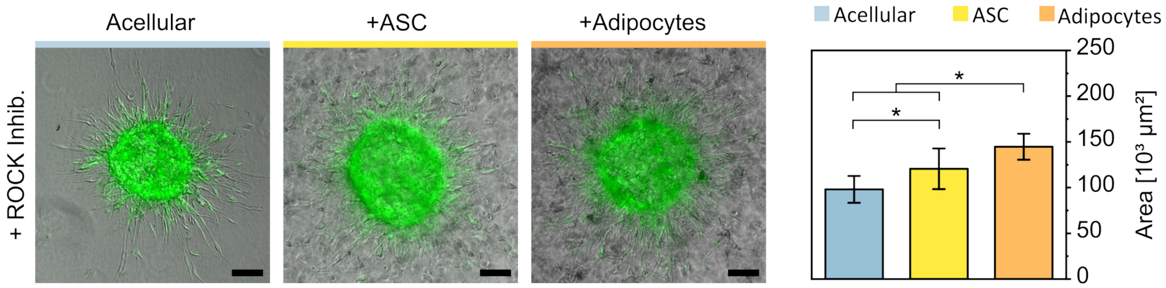

Figure S8. Analysis of MDA-MB-231 cell migration in spheroid invasion assays in dependence of ASCs or adipocytes. Phase contrast/fluorescence merge images of MDA-MB-231 spheroids and ASCs, adipocytes or no surrounding cells embedded in ColMA/HA-SH hydrogel and respective quantification of the occupied area after 44 h in culture. Data are presented as mean ± SD (n = 10-15). Significances were calculated using one-way ANOVA followed by Tukey-Kramer post hoc test (* $p < 0.05$). Scale bar: 100 µm.

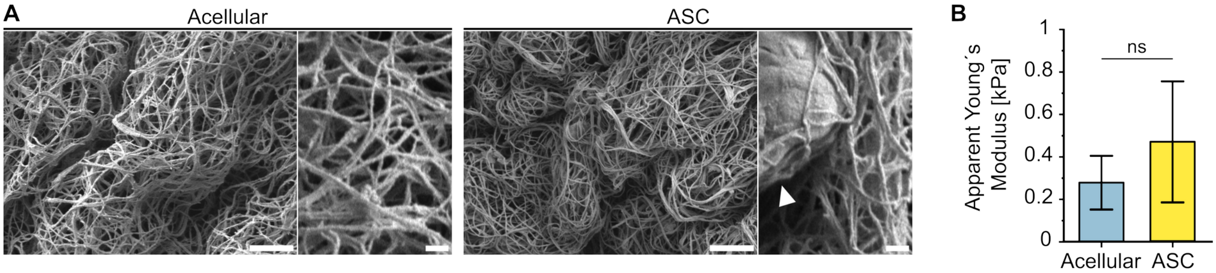

Figure S9. Analysis of ultrastructure and apparent Young's modulus of 3.5 mg mL^{-1} ColMA/HA-SH hydrogels in presence and absence of ASCs (2×10^6 cells mL^{-1}) 12 h after preparation. (A) Scanning electron microscopy images illustrating the fiber network of acellular and ASC-laden ColMA/HA-SH hydrogels. Arrowhead indicates ASC enclosed by collagen fibers. Scale bar: 400 nm and 2 µm. (B) Average apparent Young's moduli of acellular and ASC-laden ColMA/HA-SH hydrogels. For each sample, the modified one-term Ogden model was fitted to the first (unconditioned) cycle of cyclic compression data up to 35 % strain, from which the apparent Young's modulus was determined. Data are presented as mean ± SD (n = 5-6). Significances were calculated using an unpaired Student's t-test ($p < 0.05$; ns: non-significant).

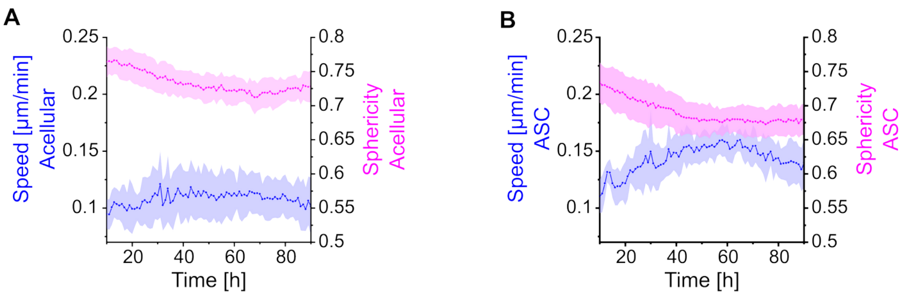

Figure S10. MDA-MB-231 cell morphology and migration speed in response to ASCs in 3D bioprinted tumor-stroma model. (A,B) Instantaneous speed and sphericity over time in tumor-acellular and in tumor-ASC model, respectively.

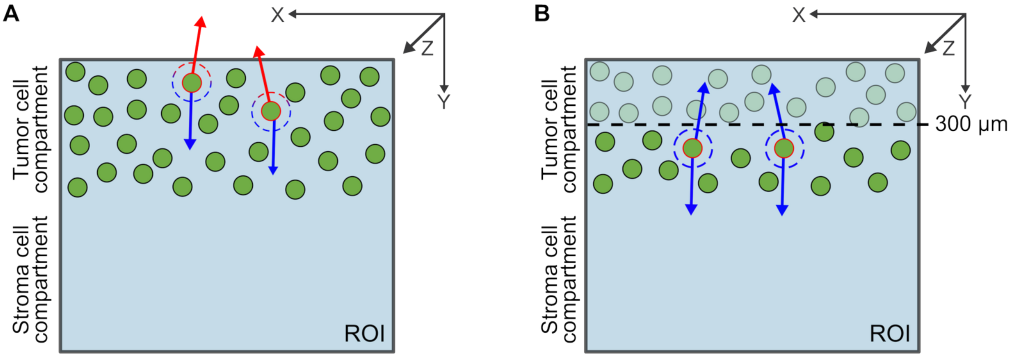

Figure S11. Analysis of the displacement toward stroma. To quantify cell displacement towards the stromal compartment (Y-Displacement), we computed the difference between the maximum Y position reached by each cell and its initial Y coordinate at t = 0 ($Y_{Displacement} = Y_{max} - Y_{t=0}$). (A) Cancer cells close to the upper tumor compartment border would not be tracked if they migrated in the negative Y direction as indicated by the red arrow. (B) Thus, only cells with an initial Y position greater than 300 µm were included in the analysis, ensuring that each cell's movement could be fully tracked in both the negative and positive Y directions.

Materials and Methods

Data Analysis and Hyperelastic Material Modeling of Cyclic Compression Tests of ColMA/HA-SH Hydrogels: To describe the deformation of ColMA/HA-SH hydrogels and to quantify the corresponding nonlinear mechanical response, we employed the nonlinear equations of continuum mechanics by introducing the deformation map $\varphi(X,t)$, which maps position X in the undeformed, unloaded configuration at time t_0, $B_0(t_0) \in R^3$, to its new position x in the deformed, loaded configuration at time t, $B_t(t)$ (**Figure S12**). The deformation gradient $F(X,t) = \nabla_X \varphi(X,t)$ maps line elements in the undeformed sample to the corresponding line elements in the deformed sample [1].

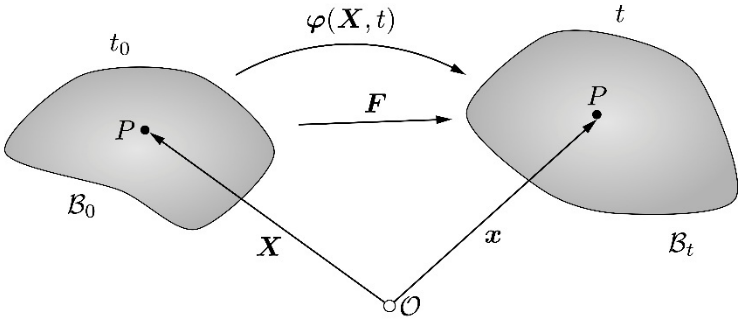

Figure S12. Kinematics of a material body in the undeformed, unloaded configuration at time t_0, $B_0(t_0) \in R^3$ and the deformed, loaded configuration at time t, $B_t(t)$.

The principal stretches λ_i, for $i=1,2,3$ describe the three-dimensional deformation state in the sample, and are the square roots of the eigenvalues of the left and right Cauchy Green strain tensors defined by $b = F F^T$ and $C = F^T F$, where $[F] = diag\{\lambda_1, \lambda_2, \lambda_3\}$.

Assuming that ColMA/HA-SH hydrogels deform homogeneously during cyclic compression, an analytical estimation of the stresses experienced by the material during the experiments was conducted. In uniaxial compression, λ_3 describes the stretch in the direction of compression as

$$\lambda_3 = 1 + \frac{\Delta z}{h} := \lambda$$

where Δz is the z-displacement and h the initial sample height. We assumed that ColMA/HA-SH hydrogels are an isotropic and incompressible material. Isotropy implies that the material properties show no significant directional dependency and thus $\lambda_1 = \lambda_2$. Incompressibility reflects the absence of volumetric changes during deformation, which is reasonable given the hydrogel´s high water content. Therefore, the incompressibility constraint ′

$$det\,F=\lambda_1\lambda_2\lambda_3=\lambda_1^2\lambda\overset{def}{=}1.$$

holds and we calculated the remaining principal stretches as $\lambda_1=\lambda_2=\lambda^{-1/2}$. This yields the deformation gradient

$$[F]=\begin{bmatrix}\lambda^{-1/2} & 0 & 0\\ 0 & \lambda^{-1/2} & 0\\ 0 & 0 & \lambda\end{bmatrix}.$$

To characterize the time-independent, elastic behavior of ColMA/HA-SH hydrogels, we used the strain energy function ψ for incompressible, hyperelastic materials

$$\psi=\psi(F)-p(J-1)$$

with the Lagrange multiplier p, which describes the hydrostatic pressure and enforces incompressibility constraint $J=det\,F=1$. To capture the unconditioned mechanical response of ColMA/HA-SH hydrogels during cyclic compression, we used the modified one-term Ogden model defined by the strain-energy function [2]

$$\psi_O(F)=\frac{2\mu}{\alpha^2}\left[\lambda_1^\alpha+\lambda_2^\alpha+\lambda_3^\alpha-3\right]$$

with the classical shear modulus μ and the nonlinearity parameter α. To calculate the stress state during cyclic compression, we introduced the Piola stress P_{zz}, which is defined as the measured force f_z per initial cross-sectional area A of the sample in the undeformed, unloaded configuration. For incompressible, hyperelastic materials, the Piola stress P is defined as the derivative of the strain-energy function ψ with respect to the deformation gradient F [1]

$$P^\psi=\frac{\partial\psi}{\partial F}=\frac{\partial\psi_O(F)}{\partial F}-p\frac{\partial J}{\partial F}=\frac{\partial\psi_O(F)}{\partial F}-pF^{-T}$$

with

$$\frac{\partial\psi_O(F)}{\partial F}=\sum_{i=1}^{3}\frac{\partial\psi(F)}{\partial\lambda_i}n_i\otimes N_i=\sum_{i=1}^{3}\frac{2\mu}{\alpha}\lambda_i^{\alpha-1}$$

where n_i and N_i are the eigenvectors of the left and right Cauchy Green strain. Considering the boundary conditions for uniaxial compression, $P_{xx}^\psi=P_{yy}^\psi=0$, we calculated the Lagrange multiplier p to

$$p=\frac{2\mu}{\alpha}\left(\lambda^{\frac{-\alpha}{2}}\right)$$

and the Piola stress in z-direction P_{zz}^ψ with $\lambda_3=\lambda$ to

$$P_{zz}^\psi=\frac{2\mu}{\alpha}\left(\lambda^{\alpha-1}-\lambda^{\frac{-\alpha}{2}-1}\right).$$

We used the nonlinear least-squares (nonlinear data-fitting) algorithm *lsqnonlin* in MATLAB version R2024b Update 3 (24.2.0.2806996) (MathWorks, Natick, MA, USA) to calibrate the classical shear modulus μ and nonlinearity parameter α by minimizing the objective function

$$\chi^2=\sum_{i=1}^{n}(¿P_{zz}-P_{zz}^{\psi})_i^2¿$$

with the number of considered experimental data points n, the experimentally determined Piola stress P_{zz} and the predicted Piola stress P_{zz}^{ψ} using the modified one-term Ogden model. To evaluate the capability of the modified one-term Ogden model to capture the time-independent, elastic response of ColMA/HA-SH hydrogels, we quantified the quality of the fitting results in terms of differences in the root mean squared error (RMSE) between experimental data and model output for compression.

We calculated the apparent Young's modulus E_{app} from the classical shear modulus μ and the initial Poisson's ratio v through the relation

$$E_{app}=2\mu(1+v).$$

Considering the incompressibility constraint, $v=0.5$ applies and we determined the apparent Young's modulus as $E_{app}=2\mu(1+0.5)=3\mu$.

RNA Isolation and Gene Expression Analysis: Constructs were homogenized in TRIzol reagent (Invitrogen, Waltham, MA, USA) for 5 min at 25 Hz (TissueLyser II, Qiagen, Hilden, Germany) followed by RNA isolation according to the manufacturer's instructions. cDNA was synthesized using the High-Capacity cDNA Reverse Transcription Kit (Applied Biosystems, Foster City, CA, USA). Subsequently, real-time RT-qPCR was performed using self-designed, intron spanning primers (see **Table 1**) and MESA GREEN qPCR Mastermix Plus for SYBR® Assay (Eurogentec, Liège, Belgium) in a CFX96 real-time system (Bio-Rad, Hercules, CA, USA). Relative gene expression of all samples was normalized to the housekeeping gene eukaryotic translation elongation factor 1 alpha (EF1α) and to the gene expression of 2.5 mg mL^{-1} ColMA samples at day 0. The $2^{-\Delta\Delta CT}$ method was used to determine the x-fold increase of expression levels of each gene [3].

Table 1: List of self-designed primers used for RT-qPCR

Primer	Sequence 5´→ 3´
EF1α forward	GCCCATGTGTGTTGAGAGC
EF1α reverse	CCGCAACTGTCTGTCTCATATC
MMP-1 forward	TTCGGGGAGAAGTGATGTTC

MMP-1 reverse	ATCTCTGTCGGCAAATTCGT
MMP-2 forward	GAAGGATGGCAAGTACGGCT
MMP-2 reverse	GCGGAATGGAAACTTGCAGG
MMP-7 forward	CGGATGGTAGCAGTCTAGGG
MMP-7 reverse	TGGGGATCTCCATTTCCATA
MMP-8 forward	ACTATGGCTTCCCCAGCAG
MMP-8 reverse	ACCTGGCTCCATGAATTGTC
MMP-9 forward	CGACGTCTTCCAGTACCGAG
MMP-9 reverse	GGGCACTGCAGGATGTCATA
MMP-11 forward	ACCGACAGAAGAGGTTCGTG
MMP-11 reverse	CTTTAGGGCCTCTGCCATC
MMP-12 forward	GGCCCTATGGAGGAAACATT
MMP-12 reverse	GCTTTCCGGATTGGTAGTCA
MMP-13 forward	TATGACATCCTGCAGTGCCC
MMP-13 reverse	GGGTCCTTGGAGTGGTCAAG
MMP-14 forward	ACACCCACTTTGACTCTGCC
MMP-14 reverse	CACAGCCACCAGGAAGATGT
MMP-15 forward	CCTGGACAACTATCCCATGC
MMP-15 reverse	AAAGAGCCAGTAGCGGTCAC
MMP-16 forward	GACCCCAGAATGTCAGTGCT
MMP-16 reverse	GGGCTTCTTCATCCAGTCAA

Analysis of Secreted MMP-1 and General Protease Activity of Conditioned Media: Supernatants of MDA-MB-231-laden hydrogels (5×10^6 cells mL^{-1}) were collected after 24 h (day 0-1) and after 48 h (day 0-2) in culture and stored at −20 °C until analysis. MMP-1 concentrations were determined via human total MMP-1 DuoSet ELISA (R&D Systems, Minneapolis, MN, USA), according to the manufacturer’s instructions. MMP-1 levels were normalized to the total DNA content of the respective constructs. Hydrogels were harvested in 300 µl phosphate/saline buffer, homogenized via TissueLyser II and sonicated. DNA content was quantified with the Quant-iT PicoGreen DNA Assay Kit (Invitrogen) according to the manufacturer’s instructions and measured fluorometrically (excitation: 480 nm, emission: 520 nm; Tecan GENios pro; Tecan, Maennedorf, Switzerland). To quantify protease activity of the supernatants, a protease reporter peptide with the sequence

K(Dnp)SGPAGLRGGSK(Mca) was synthesized via standard N-α-fluorenylmethoxy-carbonyl solid-phase peptide synthesis, according to Hamm et al. [4]. For each sample, 5 µl of 1 mM protease reporter peptide solution was added to black 96-well plates, yielding a final concentration of 50 µM in a total reaction volume of 100 µl. Filtered (pore size 0.2 µm) supernatants were added to each well simultaneously to start the reaction. The reaction was maintained at 37 °C for 180 min and the fluorescence change was measured every 120 s at 320 nm excitation and 405 nm emission (Infinite M Plex Tecan).

Analysis of MDA-MB-231 and GFP Expressing MDA-MB-231-GPP Spheroids**:** MDA-MB-231 and GFP expressing MDA-MB-231 spheroids were generated as described in the Material and Methods section. Images of the spheroids within the 3D Petri Dishes were then captured using an Olympus IX51 inverted light microscope and analyzed with a custom-written Fiji script for segmentation. For background correction, a Gaussian blur filter (sigma = 3 px) was applied. The corrected images were then converted into binary masks, followed by a series of hole-filling, dilation and erosion operations to enhance the mask. Finally, the area, roundness and solidity of the spheroids were measured using the "analyze particle" tool.

References

1. Holzapfel G.A., *Nonlinear solid Mechanics: a Continuum approach for engineering science.* Meccanica, **2002**, 37: 489–490. DOI: 10.1023/A:1020843529530.
2. Ogden R.W., *Large deformation isotropic elasticity – on the correlation of theory and experiment for incompressible rubberlike solids.* Proceedings of the Royal Society of London. A. Mathematical and Physical Sciences, **1992**, 326(1567): 565-584. DOI: 10.1098/rspa.1972.0026.
3. Livak K.J. and Schmittgen T.D., *Analysis of relative gene expression data using real-time quantitative PCR and the 2(-Delta Delta C(T)) Method.* Methods, **2001**, 25(4): 402-8. DOI: 10.1006/meth.2001.1262.
4. Hamm P., Meinel L., and Driessen M.D., *An introductory guide to protease sensitive linker design using matrix metalloproteinase 13 as an example.* ACS Biomaterials Science Engineering, **2024**, 10(6): 3693-3706. DOI: 10.1021/acsbiomaterials.4c00407.
